# Class-matched antibiotic dispensing and susceptibility transitions in recurrent urinary gram-negative bacilli

**DOI:** 10.3389/fcimb.2026.1897524

**Published:** 2026-09-28

**Authors:** Ibrahim Tawhari, Mona M. Alshahrani, Mohammed Alshehri, Ali M. Somily, Khalifa Binkhamis, Abdulah J. Alqahtani, Abdullah A. Al-Shahrani, Safia Abdullah Mohammed, Esmahan Tahtouh, Mohammed Tawhari, Yahya Shabi, Abdulah O. S. Bawazeer

**Affiliations:** 1 Department of Medicine, College of Medicine, King Khalid University, Abha, Saudi Arabia; 2 Department of Pathology, College of Medicine, King Saud University and King Saud University Medical City, Riyadh, Saudi Arabia; 3 Department of Pathology, College of Medicine, King Saud University, Riyadh, Saudi Arabia; 4 Department of Microbiology and Clinical Parasitology, College of Medicine, King Khalid University, Abha, Saudi Arabia; 5 Department of Internal Medicine, Aseer Central Hospital, Abha, Saudi Arabia; 6 Department of Medicine, College of Medicine, King Saud Bin Abdulaziz University for Health Sciences, Ministry of the National Guard Health Affairs, Riyadh, Saudi Arabia; 7 Department of Microbiology, Aseer Central Hospital, Abha, Saudi Arabia

**Keywords:** antibiotic exposure, antimicrobial resistance, antimicrobial stewardship, difficult-to-treat resistance, gram-negative bacilli, urinary tract

## Abstract

**Objectives:**

Antimicrobial resistance (AMR) among urinary Gram-negative bacilli (GNB) is rising in Saudi Arabia, but how interim antibiotic exposure relates to within-patient changes in susceptibility is not well characterized. We evaluated the association between class-matched antibiotic exposure occurring between consecutive cultures and susceptible (S)-to-non-susceptible (NS) and non-susceptible-to-susceptible transitions.

**Methods:**

In a single-center, retrospective, culture-based, pharmacy-linked paired-culture cohort at a 500-bed tertiary hospital in southern Saudi Arabia (Aseer Central Hospital [ACH]), consecutive same-patient, same-species urinary GNB cultures 14–365 days apart formed pairs, and interim exposure was defined as ACH-recorded antibiotic dispensing. For each of the seven feasible phenotypes, adjusted transition models estimated S→NS odds and separate direct models estimated NS→S odds, with supporting analyses using inverse probability weighting, cross-class models, E-values, penalized-likelihood sensitivity analyses, and exploratory pragmatic null-covariable diagnostics.

**Results:**

The primary cohort comprised 1,274 pairs from 1,078 patients, of whom 807 (74.9%) had at least one interim ACH-recorded systemic antibiotic dispensing. Class-matched dispensing was associated with higher adjusted odds of an S→NS transition for extended-spectrum cephalosporins (OR, 3.83), difficult-to-treat resistance (OR, 2.91), piperacillin–tazobactam (OR, 2.46), and fluoroquinolones (OR, 2.29), but not carbapenems (OR, 0.93). In direct models, matched dispensing was associated with lower odds of an NS→S transition, reaching significance only for piperacillin–tazobactam (OR, 0.39).

**Conclusions:**

In recurrent urinary GNB, interim class-matched hospital-recorded antibiotic dispensing was associated with within-patient S→NS transitions for several clinically important phenotypes. These observational associations do not establish treatment effects; they identify patterns for confirmation in prospective studies and may inform surveillance and stewardship evaluation.

## Introduction

Antimicrobial resistance (AMR) is among the foremost global public health threats; bacterial AMR was estimated to be directly responsible for 1.27 million deaths and associated with 4.95 million deaths worldwide in 2019, and the misuse and overuse of antimicrobials are principal drivers of this burden (World Health Organization, 2023). International frameworks have accordingly prioritized strengthened surveillance and antimicrobial stewardship as central containment strategies (World Health Organization, 2015). Gram-negative bacilli (GNB) are major contributors to drug-resistant infection. Resistance to extended-spectrum cephalosporins (ESC), carbapenems, and fluoroquinolones progressively narrows first-line options and forces reliance on less effective or more toxic reserve agents (Tamma et al., 2022). At the severe end of this spectrum, difficult-to-treat resistance (DTR), defined as non-susceptibility to all first-line β-lactams (including carbapenems) and to fluoroquinolones, has been associated with higher mortality in Gram-negative bloodstream infection (Kadri et al., 2018). Across the Arabian Peninsula and the wider Eastern Mediterranean Region, resistant GNB causing healthcare-associated infections, including extended-spectrum β-lactamase- and carbapenemase-producing organisms, are well documented (Abdalla et al., 2023; El-Saed et al., 2016; Zowawi et al., 2013), yet the regional scope and impact of resistance remain incompletely characterized (World Health Organization Regional Office for the Eastern Mediterranean). In Saudi Arabia, repeated calls have emphasized the urgent need for coordinated surveillance and stewardship (Zowawi, 2016), and recent reports describe escalating carbapenem resistance and DTR among *Klebsiella* species, including at our own center, where carbapenem resistance among *Klebsiella* rose markedly over the past decade (Shabi et al., 2026), as well as in carbapenem-resistant *K. pneumoniae* causing bloodstream infection elsewhere in the country (Alkhelaiwi et al., 2026). Most surveillance, however, characterizes resistance cross-sectionally at the population level and does not capture how the susceptibility of an individual patient’s organisms changes over time in relation to interim antibiotic exposure. Urine is a frequent source of GNB isolates, but a positive urine culture may represent asymptomatic bacteriuria or colonization rather than infection, and unnecessary antimicrobial treatment of bacteriuria is itself a recognized driver of inappropriate antibiotic use (Nicolle et al., 2019). Whether between-culture antibiotic exposure is associated with within-patient S→NS or NS→S transitions in urinary GNB has been quantified rarely, particularly in this region. To address this gap, this study aimed to characterize within-patient changes in antimicrobial susceptibility among recurrent same-species urinary Gram-negative bacilli isolates and to evaluate whether interim antibiotic exposure was associated with these changes. We specifically sought to determine the frequency of S→NS transitions between recurrent urinary isolates, determine the frequency of NS→S transitions, and assess the association between interim class-matched or phenotype-relevant antibiotic exposure and S→NS or NS→S transitions across clinically important non-susceptibility phenotypes.

## Materials and methods

### Study design and setting

This retrospective, pharmacy-linked, paired-culture cohort study was conducted at Aseer Central Hospital, a 500-bed tertiary care hospital serving the Aseer region of southern Saudi Arabia. The study evaluated transitions in class-matched antimicrobial non-susceptibility between recurrent same-species urinary Gram-negative bacilli isolates rather than symptom-defined urinary tract infection episodes.

The study was approved by the Aseer Institutional Review Board on April 16, 2025 under the protocol titled “Anti-bacterial Resistant Trend” (IRB reference F7-2-2025; National Committee of Bioethics registration number H-06-B-091), following full-board review. The approved principal investigator was Abdulah O. S. Bawazeer and the approved co-investigator was Yahya Shabi; both are authors of this report. No amendment to the approved protocol was required for the analyses reported here. The IRB approval letter is provided as a supplementary file. Because the study was retrospective and involved secondary analysis of routinely collected clinical and microbiological data without direct patient contact, individual informed consent was not obtained. All data were handled in a de-identified manner to protect patient confidentiality.

Reporting followed the Strengthening the Reporting of Observational Studies in Epidemiology (STROBE) statement (von Elm et al., 2007), the STROBE extension for antimicrobial stewardship studies (STROBE-AMS) (Tacconelli et al., 2016), the Reporting of Studies Conducted Using Observational Routinely Collected Health Data (RECORD) statement (Benchimol et al., 2015), and the RECORD extension for pharmacoepidemiology (RECORD-PE) (Langan et al., 2018).

### Data sources and study population

#### Data extraction and study period

Two institutional data sources were linked at the patient level using the medical record number. The institutional antimicrobial susceptibility surveillance database provided all positive urine culture results and associated antimicrobial susceptibility testing data. The extracted variables included demographic characteristics, specimen and culture characteristics, organism identification, susceptibility results, and admission-linked clinical information. The institutional pharmacy dispensing record provided data on systemic antibiotics dispensed during the study period. The source cohort was restricted to records from 2013 through 2024, with data availability through August 27, 2024.

#### Inclusion and exclusion criteria

Eligible cultures were urine specimens collected at ACH during the study period in inpatient, emergency department, or outpatient settings, including midstream urine, Foley catheter urine, and suprapubic aspiration. The cultures were required to yield Gram-negative bacilli (GNB), either Enterobacterales or non-fermenting GNB. Patients younger than 12 years were excluded because pediatric services were transferred from ACH to a dedicated pediatric hospital early in the study period. A further pair-level restriction to pharmacy-observable cultures, required to ascertain between-culture antibiotic exposure, is defined in the “Pair derivation” section below.

#### Pair derivation

Culture pairs were constructed from consecutive same-patient, same-species cultures separated by 14 to 365 days. The first culture defined baseline susceptibility, the inter-culture interval defined the exposure window, and the second culture defined the transition outcome (**Figure 1**). Same-patient, same-species cultures collected within 14 days were treated as a single episode, and only the earliest was retained. A pair was considered pharmacy-observable if the patient had at least one ACH pharmacy dispensing record on or before the first culture. This criterion established prior linkability to ACH pharmacy data but did not establish complete capture of antibiotics obtained outside ACH. For descriptive analyses, the first pharmacy-observable pair per patient was retained, whereas phenotype-specific regression models used the first pharmacy-observable pair per patient-species combination. Same-species pairing did not establish strain identity; without molecular typing, a transition could reflect selection within a persisting strain, replacement by another strain of the same species, or changing detection of coexisting subpopulations.

**Figure 1:**
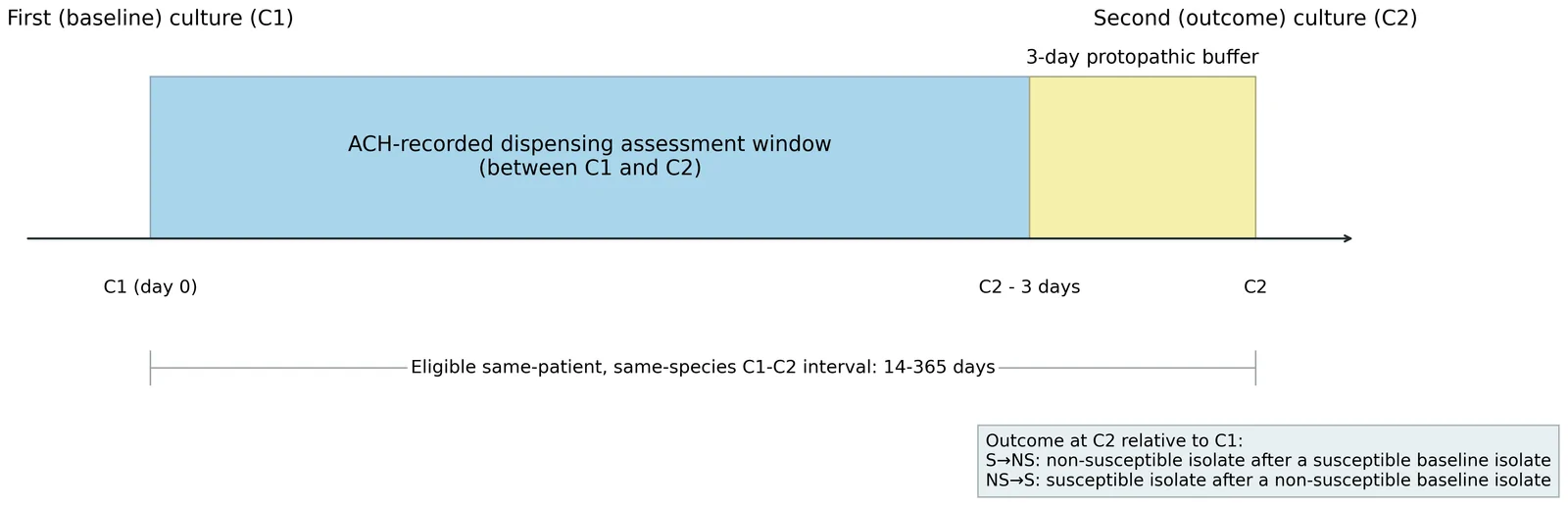
Study design and pair derivation. Pairs were defined from consecutive same-patient, same-species cultures separated by 14 to 365 days. The first culture (C1) established baseline susceptibility, ACH-recorded antibiotic dispensing was assessed between C1 and the second culture (C2), and a 3-day protopathic buffer excluded dispensing immediately before C2. Outcomes at C2 were classified relative to C1 as an S→NS transition (non-susceptible isolate after a susceptible baseline isolate) or an NS→S transition (susceptible isolate after a non-susceptible baseline isolate).

#### Between-culture exposure

Between-culture exposure was operationalized as ACH-recorded antibiotic dispensing rather than total antibiotic receipt. The institutional pharmacy record captured dispensing within ACH but not prescriptions filled at external pharmacies. An antibiotic was classified as a recorded interim exposure if it was dispensed after the first culture and before the 3-day interval immediately preceding the second culture (3-day protopathic buffer). Absence of a qualifying ACH record was classified as no ACH-recorded exposure and did not establish that the patient received no antibiotic from another source. The buffer was applied to reduce reverse causation from antibiotics prescribed in response to clinical deterioration or pending culture results immediately before the second culture. Exposures were grouped into six phenotype-matched classes: fluoroquinolones, extended-spectrum cephalosporins (ESC), carbapenems, piperacillin–tazobactam (Pip-Taz), trimethoprim–sulfamethoxazole (TMP-SMX), and aminopenicillins combined with beta-lactamase inhibitors (amino-BLI). Because difficult-to-treat resistance (DTR) is a composite phenotype without a single cognate drug class, any ACH-recorded systemic antibiotic dispensing served as its matched exposure, yielding seven exposure constructs. Clinical indication, dose, administration, adherence, and antibiotic history preceding the first culture were unavailable, and defined daily doses and prescribing care setting were not characterized. Eligible systemic routes in the dispensing record were oral, intravenous, intramuscular, and combined intravenous/intramuscular administration. Additional details on pair derivation, pharmacy observability, and exposure linkage are provided in the eMethods in the **Supplementary Material**.

### Laboratory methods

#### Organism classification and antimicrobial susceptibility testing

Organisms were classified as Enterobacterales or non-fermenting GNB: Enterobacterales were assigned according to current taxonomy (Adeolu et al., 2016), whereas non-fermenting GNB were identified by routine microbiological criteria, including failure to ferment glucose. Taxonomic nomenclature was standardized according to the List of Prokaryotic Names with Standing in Nomenclature (Parte et al., 2020). Organism identification and antimicrobial susceptibility testing were performed using the VITEK 2 automated system (bioMérieux, Marcy-l’Étoile, France). Matrix-assisted laser desorption/ionization time-of-flight mass spectrometry (MALDI–TOF MS) was not available during the study period. Minimum inhibitory concentrations (MICs) were interpreted according to the Clinical and Laboratory Standards Institute (CLSI) breakpoints in effect at the time of testing (Clinical and Laboratory Standards Institute, 2013–2024), and raw MIC values were unavailable for retrospective reinterpretation. Susceptibility results were collapsed into susceptible and non-susceptible categories, with intermediate and resistant results combined. Organism–drug pairs with intrinsic resistance, no CLSI interpretive criteria, or prespecified non-reportable combinations were suppressed before susceptibility summaries or phenotype derivation and were coded as missing rather than susceptible. A total of 23 suppression rules were executed. Because suppressed organism–drug pairs were coded as missing, they reduce the number of pairs evaluable for the affected phenotype: of the 1,274 analytic pairs, the number without a reportable result at one or both cultures was 58 for fluoroquinolones, 67 for carbapenems, 144 for piperacillin-tazobactam, 173 for trimethoprim-sulfamethoxazole, 324 for aminopenicillins combined with beta-lactamase inhibitors, 474 for difficult-to-treat resistance, and 551 for extended-spectrum cephalosporins, with the largest counts also reflecting the organism restrictions footnoted for those phenotypes (**Table 1**). These counts combine rule-based suppression with drug–organism combinations that were not tested, and the cleaned pair file does not separate the two. The evaluable pairs are, in turn, split by baseline state and therefore by transition direction in **Tables 2** and **3**.

**Table 1:** Phenotype feasibility assessment for regression analyses (*N* = 1,274 first pairs per patient-species)^a^.

Non-susceptibility phenotype	Class-matched exposure^b^	Valid pairs^c^	Susceptible at first culture	S→NS		Exposed	S→NS and exposed	Verdict^d^
				Events	Rate (%)			
Aminoglycosides^e^	Gentamicin, amikacin, or tobramycin	1,234	683	136	19.9	17	1	Excluded (matched events <10)
Carbapenems^f^	Meropenem, imipenem, or ertapenem	1,207	931	112	12.0	294	23	Included
Difficult to treat^g^	Any systemic antibiotic^h^	800	677	50	7.4	577	43	Included
Extended-spectrum cephalosporins^i^	Cefotaxime, ceftazidime, ceftriaxone, or cefepime	723	328	83	25.3	144	30	Included
Fluoroquinolones	Ciprofloxacin or levofloxacin	1,216	363	135	37.2	401	75	Included
Nitrofurantoin^j^	Nitrofurantoin	252	186	32	17.2	35	7	Excluded (matched events <10)
Aminopenicillins	Amoxicillin or ampicillin	186	43	13	30.2	6	0	Excluded (matched events <10)
Aminopenicillins + β-lactamase inhibitors	Amoxicillin–clavulanate or ampicillin–sulbactam	950	374	135	36.1	112	26	Included
Piperacillin–tazobactam	Piperacillin–tazobactam	1,130	852	123	14.4	207	41	Included
Trimethoprim–sulfamethoxazole	Trimethoprim–sulfamethoxazole	1,101	480	112	23.3	87	20	Included

NS, non-susceptible; S, susceptible.

^a^ First pharmacy-observable culture pair per patient-species (*N* = 1,274 pairs from 1,078 patients).

^b^ Class-matched antibiotic exposure dispensed between the first and second culture, excluding the 3 days before the second culture.

^c^ Valid pairs required a non-missing susceptibility result for the phenotype at both cultures.

^d^ Prespecified pragmatic gating criteria for phenotype prioritization. Included: S→NS events ≥50, exposed pairs ≥10, and matched S→NS and exposed events ≥10. Marginal: 20–49 S→NS events; reported descriptively for transparency but not entered into primary regression models. Excluded: total events <20, exposed pairs <10, or matched events <10.

^e^ Non-susceptibility to gentamicin, amikacin, or tobramycin. *Providencia stuartii*: gentamicin and tobramycin suppressed (only amikacin reportable). *Pseudomonas aeruginosa*: gentamicin suppressed (amikacin and tobramycin reportable).

^f^ Carbapenem non-susceptibility was defined as meropenem, imipenem, or ertapenem for Enterobacterales (imipenem excluded for Proteeae) and as meropenem or imipenem for *Acinetobacter* baumannii and *Pseudomonas aeruginosa*.

^g^ Organism-specific per Kadri et al. (Clin Infect Dis 2018;67:1803–14): Enterobacterales, *Acinetobacter baumannii*, and *Pseudomonas aeruginosa* only. *Stenotrophomonas maltophilia* and other non-fermenters excluded.

^h^ Difficult-to-treat non-susceptibility reflects composite non-susceptibility to all first-line agents and cannot be attributed to a single drug class. Exposure was therefore defined as any ACH-recorded dispensing of a systemic antibiotic of any class between cultures; this contrast is any versus none.

^i^ Restricted to Enterobacterales.

^j^ Restricted to Enterobacterales, excluding Proteeae and *Serratia marcescens*.

**Table 2:** Non-susceptibility state transitions by class-matched antibiotic exposure (*N* = 1,274)^a^.

Non-susceptibility phenotype	Class-matched antibiotic exposure	Transition			Risk difference, pp (95% CI)^c^
			Unexposed, *n*/*N* (%)	Exposed, *n*/*N* (%)	
Carbapenems^d^	Meropenem, imipenem, or ertapenem	S→NS	89/721 (12.3)	23/210 (11.0)	-1.39 (-5.83 to 4.01)
		NS→S	77/192 (40.1)	28/84 (33.3)	-6.77 (-18.50 to 5.82)
		Stable susceptible (S→S)	632/721 (87.7)	187/210 (89.0)	—
		Persistent non-susceptibility (NS→NS)	115/192 (59.9)	56/84 (66.7)	
Difficult-to-treat^e^	Any systemic antibiotic^f^	S→NS	7/197 (3.6)	43/480 (9.0)	+5.41 (1.25 to 8.92)
		NS→S	11/26 (42.3)	31/97 (32.0)	-10.35 (-31.32 to 9.36)
		Stable susceptible (S→S)	190/197 (96.4)	437/480 (91.0)	—
		Persistent non-susceptibility (NS→NS)	15/26 (57.7)	66/97 (68.0)	
Extended-spectrum cephalosporins^g^	Cefotaxime, ceftazidime, ceftriaxone, or cefepime	S→NS	53/266 (19.9)	30/62 (48.4)	+28.46 (15.42 to 41.54)
		NS→S	61/313 (19.5)	12/82 (14.6)	-4.85 (-12.70 to 5.19)
		Stable susceptible (S→S)	213/266 (80.1)	32/62 (51.6)	—
		Persistent non-susceptibility (NS→NS)	252/313 (80.5)	70/82 (85.4)	
Fluoroquinolones	Ciprofloxacin or levofloxacin	S→NS	60/204 (29.4)	75/159 (47.2)	+17.76 (7.71 to 27.58)
		NS→S	75/611 (12.3)	27/242 (11.2)	-1.12 (-5.57 to 4.04)
		Stable susceptible (S→S)	144/204 (70.6)	84/159 (52.8)	—
		Persistent non-susceptibility (NS→NS)	536/611 (87.7)	215/242 (88.8)	
Aminopenicillins + β-lactamase inhibitors	Amoxicillin–clavulanate or ampicillin–sulbactam	S→NS	109/316 (34.5)	26/58 (44.8)	+10.33 (-2.96 to 24.11)
		NS→S	119/522 (22.8)	15/54 (27.8)	+4.98 (-5.93 to 18.55)
		Stable susceptible (S→S)	207/316 (65.5)	32/58 (55.2)	—
		Persistent non-susceptibility (NS→NS)	403/522 (77.2)	39/54 (72.2)	
Piperacillin–tazobactam	Piperacillin–tazobactam	S→NS	82/694 (11.8)	41/158 (25.9)	+14.13 (7.40 to 21.83)
		NS→S	93/229 (40.6)	13/49 (26.5)	-14.08 (-26.52 to 0.98)
		Stable susceptible (S→S)	612/694 (88.2)	117/158 (74.1)	—
		Persistent non-susceptibility (NS→NS)	136/229 (59.4)	36/49 (73.5)	
Trimethoprim–sulfamethoxazole	Trimethoprim–sulfamethoxazole	S→NS	92/420 (21.9)	20/60 (33.3)	+11.43 (-0.02 to 24.62)
		NS→S	97/594 (16.3)	2/27 (7.4)	-8.92 (-15.43 to 7.24)
		Stable susceptible (S→S)	328/420 (78.1)	40/60 (66.7)	—
		Persistent non-susceptibility (NS→NS)	497/594 (83.7)	25/27 (92.6)	

CI, confidence interval; NS, non-susceptible; pp, percentage points; S, susceptible.

^a^ First pharmacy-observable culture pair per patient-species (*N* = 1,274 pairs from 1,078 patients).

^b^ Class-matched antibiotic exposure dispensed between the first and second culture, excluding the 3 days before the second culture.

^c^ Risk difference is expressed as exposed minus unexposed; positive values indicate higher transition probability in the exposed group. 95% CIs estimated by the Miettinen–Nurminen score method. Shown for state-changing transitions (S→NS and NS→S) only; stable susceptible and persistent non-susceptibility are complements within the same baseline-state denominator.

^d^ Carbapenem non-susceptibility was defined as meropenem, imipenem, or ertapenem for Enterobacterales (imipenem excluded for Proteeae) and as meropenem or imipenem for *Acinetobacter baumannii* and *Pseudomonas aeruginosa*.

^e^ Organism-specific per Kadri et al. (Clin Infect Dis 2018;67:1803–14): Enterobacterales, *Acinetobacter baumannii*, and *Pseudomonas aeruginosa* only. *Stenotrophomonas maltophilia* and other non-fermenters excluded.

^f^ Difficult-to-treat non-susceptibility reflects composite non-susceptibility to all first-line agents and cannot be attributed to a single drug class. Exposure was therefore defined as any ACH-recorded dispensing of a systemic antibiotic of any class between cultures; this contrast is any versus none.

^g^ Restricted to Enterobacterales.

**Table 3:** Susceptibility transitions by class-matched antibiotic exposure between consecutive cultures (*N* = 1,274)^a^.

Non-susceptibility phenotype	Class-matched exposure^b^	S→NS; odds ratio (95% CI)				NS→S^d^; odds ratio (95% CI)			
		Baseline-susceptible pairs, *n*^c^	Unadjusted^c^	Adjusted^f^	*P*-value	Baseline–non-susceptible pairs, *n*^c^	Unadjusted^e^	Adjusted^f^	*P*-value
Carbapenems^g^	Meropenem, imipenem, or ertapenem	931	0.89 (0.54 to 1.45)	0.93 (0.57 to 1.54)	0.783	276	0.58 (0.32 to 1.03)	0.62 (0.34 to 1.15)	0.132
Difficult-to-treat^h^	Any systemic antibiotic^i^	677	2.91 (1.29 to 6.56)	2.91 (1.29 to 6.56)	0.01	123	0.52 (0.21 to 1.28)	0.52 (0.20 to 1.32)	0.167
Extended-spectrum cephalosporins^j^	Cefotaxime, ceftazidime, ceftriaxone, or cefepime	328	3.74 (2.08 to 6.74)	3.83 (2.12 to 6.94)	<0.001	395	0.62 (0.32 to 1.19)	0.58 (0.30 to 1.13)	0.112
Fluoroquinolones	Ciprofloxacin or levofloxacin	363	2.27 (1.43 to 3.61)	2.29 (1.44 to 3.66)	<0.001	853	0.73 (0.46 to 1.18)	0.67 (0.41 to 1.09)	0.109
Aminopenicillins + β-lactamase inhibitors	Amoxicillin–clavulanate or ampicillin–sulbactam	374	1.56 (0.89 to 2.74)	1.58 (0.90 to 2.79)	0.114	576	1.15 (0.61 to 2.16)	1.16 (0.62 to 2.18)	0.649
Piperacillin–tazobactam	Piperacillin–tazobactam	852	2.64 (1.71 to 4.07)	2.46 (1.58 to 3.85)	<0.001	278	0.46 (0.23 to 0.93)	0.39 (0.20 to 0.79)	0.009
Trimethoprim-sulfamethoxazole	Trimethoprim–sulfamethoxazole	480	1.64 (0.89 to 3.01)	1.72 (0.92 to 3.19)	0.087	621	0.38 (0.09 to 1.63)	0.38 (0.09 to 1.65)	0.196

CI, confidence interval; NS, non-susceptible; S, susceptible.

^a^ First pharmacy-observable culture pair per patient-species. Cluster-robust standard errors (patient).

^b^ Class-matched antibiotic exposure dispensed between the first and second culture, excluding the 3 days before the second culture.

^c^ Each transition is estimated in its own baseline stratum, so the two directions have different denominators. Pairs required a non-missing susceptibility result for the phenotype at both cultures. Phenotypes were included per prespecified feasibility gating in **Table 1** (S→NS events ≥50, exposed pairs ≥10, and matched S→NS plus exposed events ≥10); that gating was applied to the S→NS outcome, and all gated-in phenotypes are retained for both directions.

^d^ NS→S odds ratios are estimated directly among baseline–non-susceptible pairs, with susceptibility at the second culture as the outcome. Baseline status and its interactions are constant or collinear in that restricted sample and are therefore omitted. Values less than 1.0 indicate that class-matched antibiotic exposure is associated with lower odds of a return to susceptibility.

^e^ Unadjusted models contain only the class-matched exposure and the inter-culture interval (restricted cubic splines, 4 knots). The S→NS model additionally retains baseline susceptibility status, its interaction with the interval, and the exposure × baseline interaction.

^f^ Same models with additional adjustment for age, sex, organism group, intensive care unit status at time of culture collection, prior culture burden, and documented comorbidity count.

^g^ Carbapenem non-susceptibility was defined as meropenem, imipenem, or ertapenem for Enterobacterales (imipenem excluded for Proteeae) and as meropenem or imipenem for *Acinetobacter baumannii* and *Pseudomonas aeruginosa*.

^h^ Organism-specific per Kadri et al. (Clin Infect Dis 2018;67:1803–14): Enterobacterales, *Acinetobacter baumannii*, and *Pseudomonas aeruginosa* only. *Stenotrophomonas maltophilia* and other non-fermenters excluded.

^i^ Difficult-to-treat non-susceptibility reflects composite non-susceptibility to all first-line agents and cannot be attributed to a single drug class. Exposure was therefore defined as any ACH-recorded dispensing of a systemic antibiotic of any class between cultures; this contrast is any versus none.

^j^ Restricted to Enterobacterales.

#### Non-susceptibility phenotype definitions

Non-susceptibility phenotypes were defined for fluoroquinolones (ciprofloxacin or levofloxacin), ESC restricted to Enterobacterales (cefotaxime, ceftazidime, ceftriaxone, or cefepime), carbapenems (ertapenem, imipenem, or meropenem), Pip-Taz, TMP-SMX, and amino-BLI (amoxicillin-clavulanate or ampicillin-sulbactam). DTR was defined separately for Enterobacterales, *Acinetobacter baumannii*, and *Pseudomonas aeruginosa* using criteria adapted from Kadri et al. (2018). *Stenotrophomonas maltophilia* and other non-fermenting GNB not represented in the Kadri framework were excluded from DTR classification. Full phenotype definitions are provided in the eMethods in the **Supplementary Material**.

#### Outcomes

The primary outcome was an S→NS transition, defined as a non-susceptible second isolate after a susceptible baseline isolate for the phenotype corresponding to the interim antibiotic class. The secondary outcome was an NS→S transition, defined as a susceptible second isolate after a non-susceptible baseline isolate. These culture-based state changes do not establish when resistance arose, whether a resistant subpopulation was already present, or whether the second isolate represented the same strain. Both directions were evaluated separately for each of the seven prespecified phenotypes. Before modeling, a phenotype was retained if the pair dataset contained at least 50 S→NS events overall, at least 10 pairs with matched antibiotic dispensing, and at least 10 S→NS events among matched-exposed pairs.

### Statistical analysis

Categorical variables were summarized as frequencies (percentages) and continuous variables as medians (interquartile range [IQR]).

For each of the seven included phenotypes, the S→NS odds ratio was estimated from a first-order Markov transition logistic model of second-culture non-susceptibility. The model included baseline susceptibility status, class-matched ACH-recorded dispensing, the inter-culture interval modeled with restricted cubic splines using four knots, and interactions of baseline susceptibility status with exposure and the interval. The exposure effect among baseline-susceptible pairs yielded the S→NS odds ratio. NS→S odds ratios were estimated directly in separate logistic models restricted to baseline-non-susceptible pairs, with susceptibility at the second culture coded as the event. The unadjusted models retained exposure and the applicable interval terms; the adjusted models added age, sex, organism group, intensive care unit status at culture collection, prior culture burden (the number of earlier urinary cultures contributed by the same patient), and a documented comorbidity count. That count was the unweighted sum of 18 documented binary conditions (acute kidney injury, asthma, cancer, chronic kidney disease, chronic liver disease, chronic obstructive pulmonary disease, congestive heart failure, dementia, diabetes, epilepsy, HIV, hypertension, ischemic heart disease, obesity, peripheral vascular disease, sickle cell disease, systemic lupus erythematosus, and stroke) and is not a validated comorbidity index. Component conditions were ascertained from admission diagnosis coding, the operative record, the pharmacy record, and the pathology reports. Admission-derived flags were episode-level, operation and pharmacy evidence was restricted through each culture, and pathology evidence was undated and assigned at the patient level; therefore, the count was not uniformly measured before the first culture. Standard errors were clustered at the patient level.

To assess sparse-data and model-performance concerns, Firth penalized-likelihood sensitivity models were fit for both transition directions and all seven phenotypes (Firth, 1993; Heinze and Schemper, 2002). These fits used profile penalized-likelihood CIs without cluster-robust variance and were compared with the conventional patient-clustered models, which remained the reference. Model N, transition events, full-model degrees of freedom, events per full-model degree of freedom, c-statistics, Brier scores, and calibration intercepts and slopes were reported (van Smeden et al., 2016; Van Calster et al., 2019). Optimism was estimated from 500 patient-cluster bootstrap resamples per model. Benjamini–Hochberg correction was applied once across the 14 conventional primary transition tests.

As a secondary analysis using an alternative confounding-adjustment approach, stabilized inverse probability weighting was applied for each phenotype. The propensity model regressed the probability of receiving the class-matched exposure on the primary-analysis covariables, baseline susceptibility status at the first culture, the inter-culture interval modeled as a restricted cubic spline, and the interval-by-baseline-susceptibility interaction. Stabilized weights used the marginal exposure probability calculated within the phenotype-specific propensity estimation sample as the numerator and the fitted probability from the full propensity model as the denominator (Cole and Hernán, 2008). Weight means, dispersion, and maxima were summarized before and after winsorization at the 1st and 99th percentiles, together with the number and percentage of weights modified. Propensity-model discrimination was summarized with the area under the receiver operating characteristic curve. The propensity-sample effective sample size was calculated over the propensity estimation sample and is not the effective *N* of the weighted outcome model. Covariable balance before and after weighting was reported for each phenotype, with the absolute standardized mean difference (SMD) and |SMD| <0.10 as the prespecified balance criterion (Austin and Stuart, 2015).

A total of 49 first-order Markov transition logistic models were fit using each antibiotic class as the exposure and each non-susceptibility phenotype as the outcome. Class-matched contrasts corresponded to the primary analysis, whereas cross-class contrasts were treated as exploratory. Benjamini–Hochberg false discovery rate correction was applied to the exploratory cross-class tests (Benjamini and Hochberg, 1995).

Three sensitivity analyses addressed the exposure window, unmeasured confounding, and covariables without a prespecified antibiotic pathway. First, the protopathic buffer was varied from the primary 3-day window to 0, 7, and 14 days, and both transition models were refit at each buffer. Second, E-values were computed for the point estimate and the CI bound closer to the null for all 14 conventional primary estimates (VanderWeele and Ding, 2017). The conversion was keyed to the overall target-transition rate in each phenotype-direction estimation sample: where that rate was below 15%, the odds ratio was used as an approximation to the risk ratio; where it was 15% or greater, the square root of the odds ratio was used (VanderWeele, 2017). Third, documented hypertension and documented diabetes were entered individually alongside class-matched exposure as pragmatic null covariables (Lipsitch et al., 2010). For each of these tests, the documented comorbidity count was recomputed leave-one-out, removing the tested condition’s own contribution so that the model did not condition on a count containing the variable under test. A result was descriptively classified as consistent with the null when the odds ratio was between 0.80 and 1.25 and the *P*-value exceeded.05; this descriptive threshold was not an equivalence test.

Three further sensitivity analyses addressed the comorbidity covariable and the analytic population. First, all 14 conventional models were refit with the documented comorbidity count omitted, leaving the remaining adjustment set and the estimation samples unchanged. Second, because pathology-derived comorbidity evidence lacked reliable dates, every count component that could have been supported by biopsy evidence was removed, and the conventional and weighted models were refit. Third, the already selected analytic pairs were restricted to those whose first culture linked to a hospital admission episode, without substituting any replacement pair; this restriction changes the target population toward admission-linked care and does not establish present-on-admission status for any condition.

The timing of model covariables was audited. Age, sex, organism group, intensive care unit status, prior culture burden, and baseline susceptibility were anchored at or before the first culture, and the inter-culture interval was classified as a pair-level follow-up design variable rather than a baseline covariable. The documented comorbidity count was of mixed timing and is not established as a uniformly pre-first-culture measurement: admission-derived flags are episode-level and can carry documentation recorded after the first culture, operation and pharmacy evidence was restricted through each culture, and pathology evidence was undated and assigned at the patient level.

In a reviewer-requested exploratory analysis, a study-defined seven-category multi-class non-susceptibility outcome was defined as non-susceptibility in at least three of seven observed categories when at least three were tested. Among pairs below that threshold at the first culture, separate patient-clustered logistic models examined any ACH-recorded dispensing, number of distinct antibiotic classes, and number of days with a dispensing record. Benjamini–Hochberg correction was applied across the three primary global tests. A panel-comparability sensitivity analysis required at least five susceptibility categories to be observed at both cultures and recomputed the outcome only from the shared panel. This exploratory outcome was not a consensus multidrug-resistance definition.

Analyses were restricted to complete susceptibility data for the phenotype under study; no imputation was performed. Because each phenotype and each transition direction has its own complete-case subset, the estimation-sample pair and event counts are reported by phenotype and transition direction in **Table 2** and **3**, alongside the exposed and unexposed subsets contributing to each contrast; feasibility-gating counts are shown in **Table 1**. A nominal two-sided *P* <.05 was used before multiplicity correction; false-discovery-rate-adjusted *q*-values were used for the defined multiplicity families. Data were analyzed from March 19 to July 29, 2026 using Stata, version 19.5 (StataCorp LLC), R, version 4.6.1 (R Foundation for Statistical Computing), and Python, version 3.14.6 (Python Software Foundation). Miettinen–Nurminen risk differences used the Stata module rdcii, version 1.2, with the ridder root finder, version 1.1.2; E-values used the Stata module evalue, version 1.3.0 (Linden et al., 2020); and distinct patient counts used distinct, version 1.2.1. Firth penalized-likelihood models used the R package logistf, version 1.26.1, and c-statistics used pROC, version 1.19.0.1. Antibiotic-dispensing linkage used pandas, version 3.0.3, with numpy, version 2.5.0, and pyarrow, version 24.0.0. Additional details on model specification, weighting, and sensitivity analyses are provided in the eMethods in the **Supplementary Material**. Results required for interpretation of the primary estimates are reported in the main text; per-model diagnostics, full cross-class matrices, and buffer-specific estimates are retained in the **Supplementary Material** for length.

## Results

### Study population

The institutional antimicrobial susceptibility surveillance database contained 54,111 isolates from 22,472 patients collected between 2012 and 2024, including 19,497 urine specimens. After excluding 643 cultures from pediatric patients, 3,944 cultures yielding non-Gram-negative organisms, 105 external cultures, 218 cultures outside the study period (January 1, 2013 to August 27, 2024), and 1,047 same-patient, same-species duplicate cultures were collected within 14 days; 13,540 urinary GNB cultures from 8,045 unique patients remained (**Figure 2**). Among these cultures, 3,285 (24.3%) had a subsequent culture yielding the same species, and 2,743 (83.5% of candidate same-species re-cultures) occurred within the prespecified 14- to 365-day window for pair construction. Of these window-eligible pairs, 2,423 (88.3%) met the pharmacy-observability criterion and were eligible for analyses of ACH-recorded interim dispensing. Restricting the analysis to the first pharmacy-observable pair for each patient-species combination yielded the primary analytic cohort of 1,274 pairs from 1,078 unique patients. The baseline characteristics of the 1,078 patients are shown in **Table 4**. The median age was 61 years (IQR, 40–76), 573 (53.2%) were men, and hypertension (380 [35.3%]) and diabetes (213 [19.8%]) were the most frequent comorbidities; the three most common organisms were *Escherichia coli* (578 [53.6%]), *Klebsiella pneumoniae* (292 [27.1%]), and *Pseudomonas aeruginosa* (86 [8.0%]), and the median interval between cultures was 59 days (IQR, 29– 140). Overall, 807 patients (74.9%) had at least one ACH-recorded systemic antibiotic dispensing between cultures, most commonly fluoroquinolones (359 [44.5% of exposed patients]), carbapenems (255 [31.6%]), ESC (251 [31.1%]), Pip-Taz (194 [24.0%]), amino-BLI (112 [13.9%]), and TMP-SMX (80 [9.9%]); among the exposed patients, the median number of distinct classes was 2 (IQR, 1–3) over a median of 4 days (IQR, 1–9). Cultures were obtained in the emergency department (422 [39%]), inpatient wards (549 [51%], including 122 [22.2%] in the intensive care unit), and outpatient clinics (107 [10%]); 271 patients had no between-culture ACH-recorded dispensing, the documented comorbidity count had a median of 0 (IQR, 0–1), and baseline non-susceptibility ranged from 114 of 832 valid pairs (13.7%) for DTR to 728 of 1,050 (69.3%) for fluoroquinolones (**Supplementary Tables S1, S2**).

**Figure 2:**
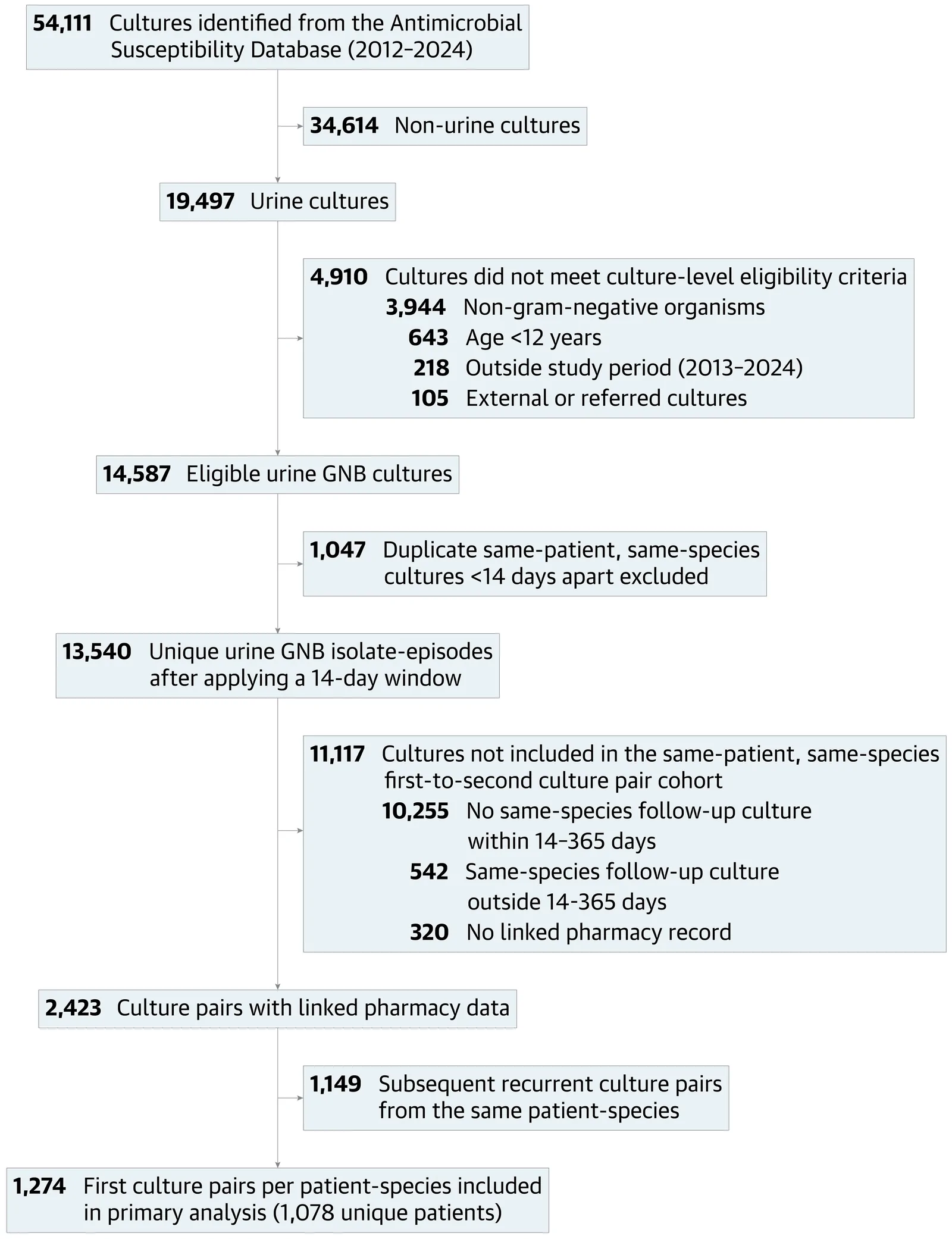
Study flowchart. Boxes from 54,111 down to 13,540 are at the culture level; boxes from 2,423 down to 1,274 are at the culture-pair level. The final analytic cohort comprises 1,274 first patient-species culture pairs from 1,078 unique patients.

**Table 4:** Characteristics of the descriptive cohort with same-species urinary GNB reculture (*N* = 1,078)^a^.

Characteristics	Overall (*N* = 1,078)	Between-culture ACH-recorded antibiotic dispensing	
		No ACH-recorded dispensing (*n* = 271)	Any ACH-recorded dispensing (*n* = 807)
Patient characteristics			
Age, years, median (IQR)	61 (40–76)	63 (41–78)	61 (39–76)
Sex, n (%)			
Men	573 (53.2)	136 (50.2)	437 (54.2)
Women	505 (46.8)	135 (49.8)	370 (45.8)
Comorbidities, n (%)^b^			
Cancer (any)	77 (7.1)	14 (5.2)	63 (7.8)
Congestive heart failure	16 (1.5)	1 (0.4)	15 (1.9)
Chronic kidney disease	62 (5.8)	12 (4.4)	50 (6.2)
Chronic obstructive pulmonary disease	6 (0.6)	1 (0.4)	5 (0.6)
Diabetes mellitus	213 (19.8)	50 (18.5)	163 (20.2)
Hypertension	380 (35.3)	90 (33.2)	290 (35.9)
Obesity	5 (0.5)	1 (0.4)	4 (0.5)
Stroke	42 (3.9)	6 (2.2)	36 (4.5)
Documented comorbidity count, median (IQR)^l^	0 (0–1)	0 (0–1)	0 (0–1)
Setting and interval			
Emergency department, n (%)	422 (39.1)	168 (62.0)	254 (31.5)
Inpatient, n (%)	549 (50.9)	66 (24.4)	483 (59.9)
Intensive care unit, n (%)^c^	122 (22.2)	30 (45.5)	92 (19.0)
Outpatient, n (%)	107 (9.9)	37 (13.7)	70 (8.7)
Inter-culture interval, days, median (IQR)	59 (29–140)	57 (28–117)	63 (30–147)
Organism, *n* (%)^d^			
* Acinetobacter baumannii*	24 (2.2)	5 (1.8)	19 (2.4)
* Enterobacter cloacae*	15 (1.4)	7 (2.6)	8 (1.0)
* Escherichia coli*	578 (53.6)	149 (55.0)	429 (53.2)
* Klebsiella pneumoniae*	292 (27.1)	62 (22.9)	230 (28.5)
* Proteus mirabilis*	47 (4.4)	11 (4.1)	36 (4.5)
* Providencia stuartii*	8 (0.7)	2 (0.7)	6 (0.7)
* Pseudomonas aeruginosa*	86 (8.0)	27 (10.0)	59 (7.3)
Other^e^	28 (2.6)	8 (3.0)	20 (2.5)
Baseline non-susceptibility, *n*/*N* (%)^f^			
Carbapenems^g^	227/1,050 (21.6)	46/261 (17.6)	181/789 (22.9)
Difficult to treat^h^	114/832 (13.7)	23/215 (10.7)	91/617 (14.7)
Extended-spectrum cephalosporins^i^	406/748 (54.3)	99/194 (51.0)	307/554 (55.4)
Fluoroquinolones^j^	728/1,050 (69.3)	181/263 (68.8)	547/787 (69.5)
Aminopenicillins + β-lactamase inhibitors^k^	522/878 (59.5)	114/201 (56.7)	408/677 (60.3)
Piperacillin–tazobactam	236/998 (23.6)	62/244 (25.4)	174/754 (23.1)
Trimethoprim–sulfamethoxazole	527/959 (55.0)	128/237 (54.0)	399/722 (55.3)

GNB, Gram-negative bacilli; IQR, interquartile range.

^a^ Descriptive cohort: First pharmacy-observable culture pair per patient (*N* = 1,078). Exposure groups indicate ACH-recorded dispensing between cultures; no qualifying record does not exclude antibiotics obtained elsewhere. The primary analytic cohort for regression analyses retained the first pharmacy-observable pair per patient-species combination (*N* = 1,274 pairs from 1,078 unique patients; see **Table 1**).

^b^ Selected comorbidities are shown; the full profile and the 18 components of the documented comorbidity count appear in **Supplementary Table S1**.

^c^ Percentages among inpatients (*N* = 549 overall; 66 with no ACH-recorded dispensing and 483 with ACH-recorded dispensing).

^d^ Species frequency by year in **Supplementary Table S2**.

^e^ Includes Gram-negative bacilli not among the seven most common species.

^f^ Patient-level denominators (descriptive cohort, *N* = 1,078 patients; first pharmacy-observable pair per patient). Each phenotype’s denominator is the count of patients whose first culture had a Clinical and Laboratory Standards Institute reportable result for that drug class; cultures failing CLSI reportability are excluded from that phenotype’s row. Pair-level baseline counts for the primary analytic cohort (*N* = 1,274 pairs) are shown in **Table 1**.

^g^ Carbapenem non-susceptibility was defined as meropenem, imipenem, or ertapenem for Enterobacterales (imipenem excluded for Proteeae) and as meropenem or imipenem for *Acinetobacter baumannii* and *Pseudomonas aeruginosa*.

^h^ Organism-specific per Kadri et al. (Clin Infect Dis 2018;67:1803–14): Enterobacterales, *Acinetobacter baumannii*, and *Pseudomonas aeruginosa* only. *Stenotrophomonas maltophilia* and other non-fermenters excluded.

^i^ Cefotaxime, ceftazidime, ceftriaxone, or cefepime; restricted to Enterobacterales.

^j^ Ciprofloxacin or levofloxacin.

^k^ Amoxicillin–clavulanate or ampicillin–sulbactam.

^l^ The documented comorbidity count sums 18 binary conditions found in available clinical records. It is not a validated index, and some conditions could not be confirmed as present before the first culture. Absence of a qualifying record was treated as absence of the condition.

### Susceptible-to-non-susceptible transitions

Of the 10 candidate phenotypes, seven met the prespecified feasibility criteria and were retained for primary modeling: carbapenems, difficult-to-treat resistance, extended-spectrum cephalosporins, fluoroquinolones, aminopenicillins combined with beta-lactamase inhibitors, piperacillin–tazobactam, and trimethoprim–sulfamethoxazole; aminoglycoside, nitrofurantoin, and aminopenicillin-only phenotypes did not meet the feasibility criteria (**Table 1**).

Among baseline-susceptible pairs, S→NS transitions were more frequent in matched-exposed than unexposed pairs for ESC (48.4% vs. 19.9%; risk difference [RD], 28.5 percentage points [95% CI, 15.4 to 41.5]), fluoroquinolones (47.2% vs. 29.4%; RD, 17.8 [7.7 to 27.6]), Pip-Taz (25.9% vs. 11.8%; RD, 14.1 [7.4 to 21.8]), and DTR (9.0% vs. 3.6%; RD, 5.4 [1.3 to 8.9]); differences were imprecise for TMP-SMX (33.3% vs. 21.9%; RD, 11.4 [–0.02 to 24.6]) and amino-BLI (44.8% vs. 34.5%; RD, 10.3 [–3.0 to 24.1]), and the carbapenem S→NS transition rate was similar between groups (11.0% vs. 12.3%) (**Table 2**). After adjustment, matched ACH-recorded dispensing was associated with higher odds of an S→NS transition for ESC (OR, 3.83; 95% CI, 2.12 to 6.94; *P* <.001), DTR (OR, 2.91; 95% CI, 1.29 to 6.56; *P* = .010), Pip-Taz (OR, 2.46; 95% CI, 1.58 to 3.85; *P* <.001), and fluoroquinolones (OR, 2.29; 95% CI, 1.44 to 3.66; *P* <.001). Associations were imprecise for TMP-SMX (OR, 1.72; 95% CI, 0.92 to 3.19; *P* = .087), amino-BLI (OR, 1.58; 95% CI, 0.90 to 2.79; *P* = .114), and carbapenems (OR, 0.93; 95% CI, 0.57 to 1.54; *P* = .783) (**Table 3**, **Figure 3**). Because DTR is a composite phenotype with no single cognate class, its matched exposure was any systemic antibiotic dispensing; that contrast is therefore any versus no ACH-recorded antibiotic rather than a class-specific comparison.

**Figure 3:**
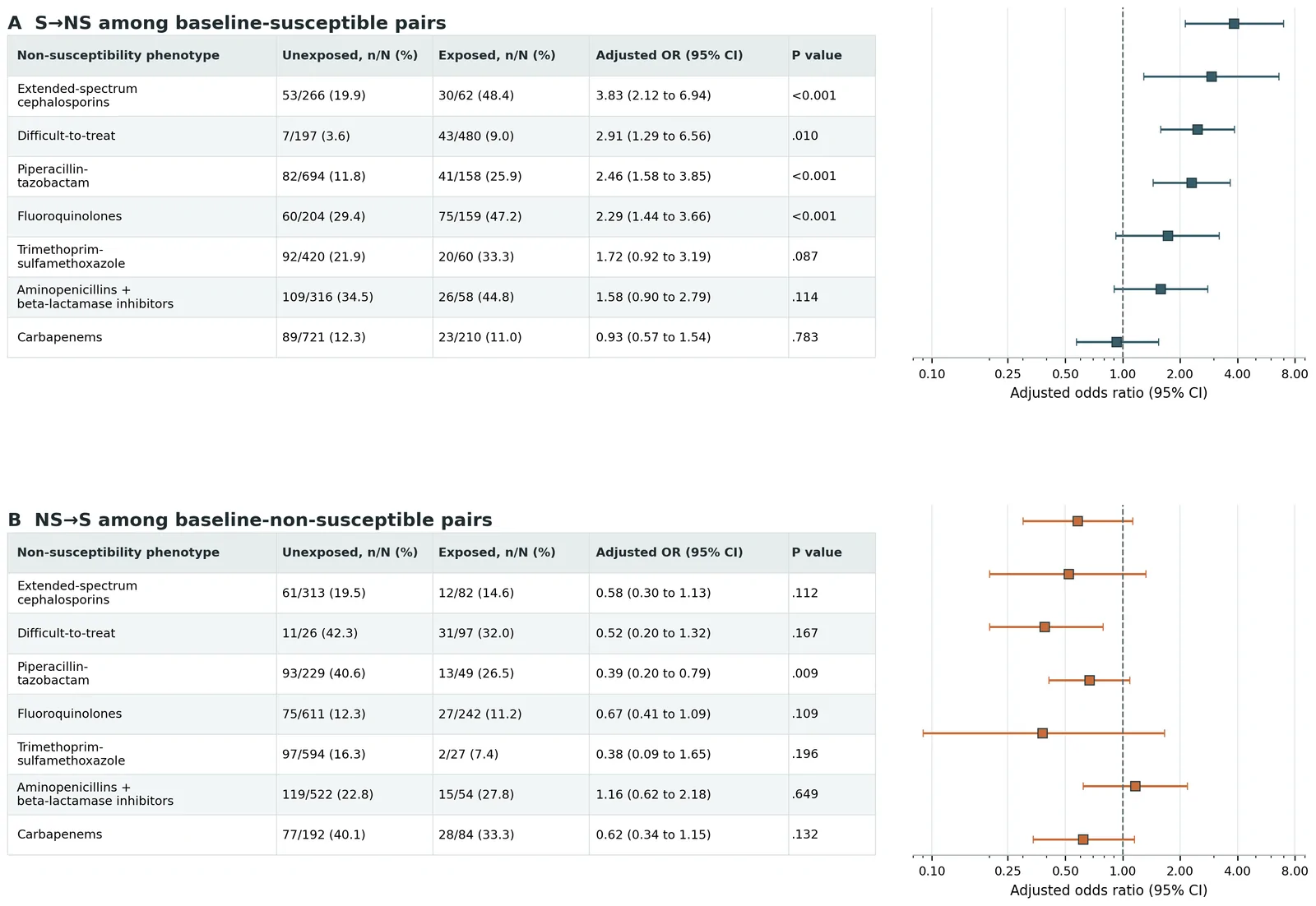
Primary S→NS and NS→S associations. **(A)** Observed rates and adjusted odds ratios for matched ACH-recorded dispensing among baseline-susceptible pairs (S→NS). **(B)** Corresponding results among baseline-non-susceptible pairs (NS→S). Observed transition rates are unadjusted proportions, whereas odds ratios are derived from adjusted direction-specific models. *P*-values correspond to the adjusted model.

### Non-susceptible-to-susceptible transitions

In direct models restricted to baseline-non-susceptible pairs, matched ACH-recorded dispensing was associated with lower odds of an NS→S transition for Pip-Taz (OR, 0.39; 95% CI, 0.20 to 0.79; *P* = .009) (**Table 2**, **Figure 3**, **Supplementary Figure S1**). The estimates were also below 1.0 but imprecise for carbapenems (OR, 0.62; 95% CI, 0.34 to 1.15; *P* = .13), DTR (OR, 0.52; 95% CI, 0.20 to 1.32; *P* = .17), ESC (OR, 0.58; 95% CI, 0.30 to 1.13; *P* = .11), fluoroquinolones (OR, 0.67; 95% CI, 0.41 to 1.09; *P* = .11), and TMP-SMX (OR, 0.38; 95% CI, 0.09 to 1.65; *P* = .20); amino-BLI showed no clear association (OR, 1.16; 95% CI, 0.62 to 2.18; *P* = .65). The Pip-Taz NS→S association remained significant after correction across the 14 primary tests (*q* = .028). Absolute modeled transition probabilities varied by phenotype and nonlinearly across the 14- to 365-day inter-culture interval. NS→S probabilities generally increased with longer intervals but remained lower in matched-exposed than unexposed pairs for most phenotypes, whereas S→NS curves were comparatively stable for some phenotypes and nonlinear for others. The reported odds ratios should therefore be interpreted as adjusted exposure contrasts from direction-specific models, not as constant absolute risk differences across follow-up (**Figure 4**).

**Figure 4:**
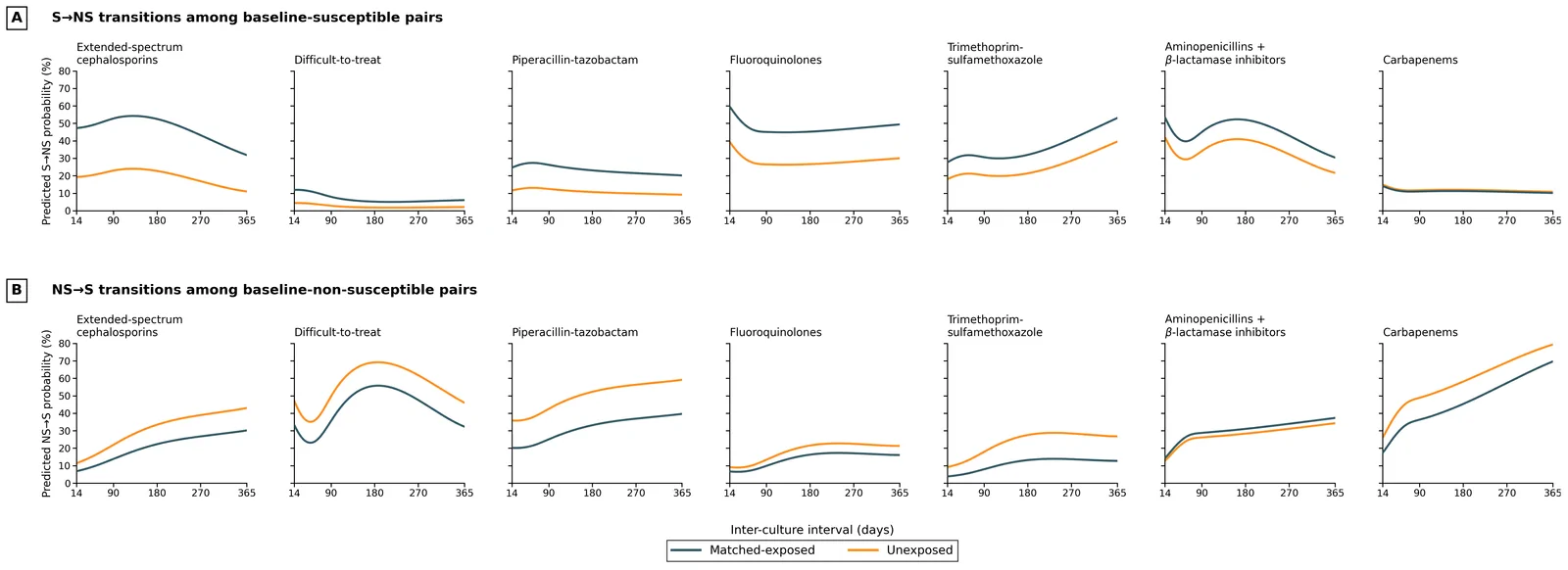
Modeled transition probabilities across the inter-culture interval. **(A)** Predicted S→NS probabilities among baseline-susceptible pairs. **(B)** Predicted NS→S probabilities among baseline-non-susceptible pairs. Within each panel, matched-exposed and unexposed curves are shown separately for each retained phenotype over the observed 14- to 365-day interval; curves are point predictions from the direction-specific adjusted transition models.

### Secondary and sensitivity analyses

After Benjamini–Hochberg correction across the single family of 14 primary transition tests, five associations remained significant: four in the S→NS direction, for ESC (*q* = .00013), Pip-Taz (q = .00054), fluoroquinolones (*q* = .00241), and DTR (*q* = .028), together with Pip-Taz in the NS→S direction (*q* = .028). The remaining nine tests did not reach significance after correction (all *q* ≥.05).

The 14 conventional transition models contained 3.3 to 13.4 target-transition events per full-model degree of freedom, and 10 of the 14 fell below 10 events per degree of freedom. Apparent c-statistics ranged from 0.578 to 0.689 and optimism-corrected c-statistics from 0.531 to 0.650. Optimism-corrected Brier scores ranged from 0.068 to 0.248, calibration intercepts from -0.030 to 0.009, and calibration slopes from 0.413 to 0.805, indicating appreciable optimism in the fitted probabilities; these models are reported as exposure-contrast estimators rather than validated prediction models. All 500 patient-cluster bootstrap resamples succeeded for every model. All 14 Firth sensitivity models converged. Their directions and inferential conclusions were consistent with the conventional patient-clustered models; for Pip-Taz NS→S, the Firth estimate was 0.42 (95% CI, 0.19 to 0.85; *P* = .016) compared with 0.39 (95% CI, 0.20 to 0.79) in the reference model. Firth inference used unclustered profile penalized likelihood, so the patient-clustered conventional estimates remained the reference.

Inverse probability weighting produced S→NS estimates similar in magnitude to the primary results across all seven phenotypes (**Supplementary Figure S2**). The four significant primary associations remained materially unchanged after weighting. The weighted TMP-SMX estimate moved upward, but its 95% CI still crossed 1.0 (OR, 1.92; 95% CI, 0.99 to 3.74; *P* = .054), so weighting did not change that inference. Propensity-model areas under the receiver operating characteristic curve ranged from 0.610 to 0.702. Before winsorization, mean weights ranged from 0.996 to 1.005 and maxima from 2.43 to 5.83; after winsorization, mean weights ranged from 0.989 to 0.997 and maxima from 1.53 to 2.25. Winsorization modified 16 to 24 weights per phenotype (1.8% to 2.0%), and propensity-sample effective sample sizes ranged from 89.7% to 97.8% of the corresponding propensity samples. Across the 49 post-weighting balance checks, two residual SMD cells reached or exceeded 0.10, both in the age and sex covariables (Pip-Taz age, 0.115; TMP-SMX sex, 0.102); all post-weighting absolute SMDs were below 0.12.

In exploratory cross-class models, the full diagonal and off-diagonal estimates and their separately corrected *q*-values are shown in **Figure 5**. Buffer-specific estimates from 0-, 3-, 7-, and 14-day protopathic windows are shown in **Supplementary Figure S3**.

**Figure 5:**
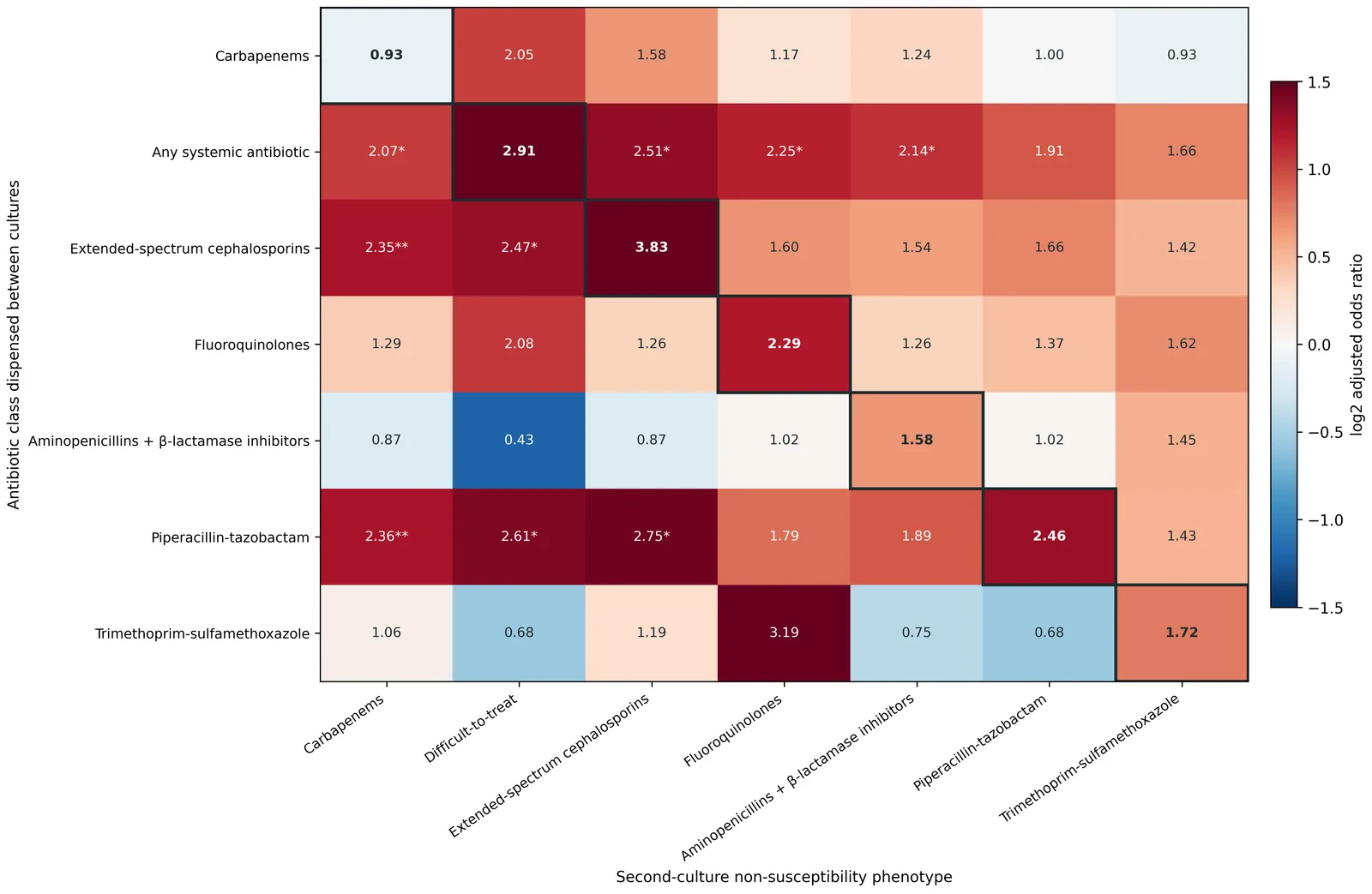
Cross-class non-susceptibility heatmap. Rows represent antibiotic exposure classes and columns represent second-culture non-susceptibility phenotypes. Each cell shows the adjusted odds ratio for an S→NS transition from a separate first-order Markov transition logistic model; bold diagonal cells are the class-matched primary analyses, and off-diagonal cells are exploratory cross-class tests after Benjamini–Hochberg correction (*q <.05; **q <.01). *q*, false-discovery-rate-adjusted *P*-value.

Using the transition-frequency-appropriate odds-ratio conversion, the point-estimate and CI-bound E-values for the five associations that survived primary-family correction were 5.26 and 1.90 for DTR S→NS, 3.33 and 2.27 for ESC S→NS, 2.40 and 1.69 for fluoroquinolone S→NS, 4.36 and 2.53 for Pip-Taz S→NS, and 2.57 and 1.50 for Pip-Taz NS→S (**Supplementary Figure S4**). E-values address unmeasured confounding under the stated conversion assumptions only; they do not address selection, exposure or outcome misclassification, or model misspecification.

The covariable-timing audit confirmed that all designated baseline variables other than the documented comorbidity count were anchored at or before the first culture; the inter-culture interval was a follow-up design variable determined by the second culture. In the no-count sensitivity analysis, all 14 conventional models were refit on estimation samples identical to the primary models; the largest absolute change in any odds ratio was 0.134 (Pip-Taz S→NS, 2.464 to 2.598), and no significance conclusion changed. Biopsy-derived components contributing to the documented comorbidity count occurred in 30 of 1,274 pairs from 28 patients. In the biopsy sensitivity analysis, conservatively excluding every potentially biopsy-supported component changed the conventional odds ratios by no more than 0.018 and no estimate, including those from the weighted models, by more than 0.034, and no significance conclusion changed; exclusive source provenance could not be identified from the cleaned pair file.

In the admission-linked sensitivity analysis, retaining only those already selected pairs whose first culture linked to an admission episode left 646 of 1,274 pairs from 554 patients. Moreover, 12 of the 14 adjusted models were reportable, including two S→NS fits affected by separation in off-target nuisance parameters in which every target pair was retained. All 12 kept the direction of the corresponding primary estimate, and only the ESC and Pip-Taz S→NS associations remained nominally significant. The amino-BLI NS→S model was not estimable because of nuisance-parameter separation, and the TMP-SMX NS→S model was not estimable because no exposed transitions remained. This restriction changes the target population and does not establish present-on-admission status.

The 14 leave-one-out phenotype-null-covariable combinations yielded 28 converged transition-model estimates, of which 15 met the descriptive null criterion and 13 departures fell outside it. The S→NS direction contributed nine results meeting the criterion and five departures, and the NS→S direction contributed six and eight, respectively. The S→NS departures were documented diabetes in the DTR, ESC, and Pip-Taz models and documented hypertension in the fluoroquinolone and TMP-SMX models; the NS→S departures were documented diabetes in the ESC, fluoroquinolone, amino-BLI, and TMP-SMX models and documented hypertension in the carbapenem, fluoroquinolone, Pip-Taz, and TMP-SMX models. Both null covariables met the criterion for S→NS in the carbapenem and amino-BLI models and for NS→S in the DTR models only. Only two departures had a *P*-value below.05: documented diabetes in the Pip-Taz S→NS model (OR, 0.49; 95% CI, 0.29 to 0.83; *P* = .008) and documented diabetes in the fluoroquinolone NS→S model (OR, 1.99; 95% CI, 1.14 to 3.46; *P* = .015); the remaining 11 departed on the odds-ratio bound alone, with *P*-values from.057 to.44. These were exploratory pragmatic null-covariable diagnostics rather than validated negative-control exposures, and departures from the descriptive threshold were not interpreted as proof of confounding.

Among 623 pairs below the study-defined multi-class non-susceptibility threshold at the first culture, 161 crossed the threshold at the second culture. Any ACH-recorded dispensing was not clearly associated with that transition (OR, 1.40; 95% CI, 0.91 to 2.16; *P* = .126; *q* = .126). This any-antibiotic contrast addresses a different outcome from the DTR transition model, and the two are not directly comparable. Among the 459 pairs with recorded dispensing (126 events), the global associations differed by number of distinct classes (*P* = .0063; *q* = .0096) and number of days with a dispensing record (*P* = .0064; *q* = .0096). Compared with one class, the ORs were 2.37 (95% CI, 1.36 to 4.11) for two classes and 1.86 (95% CI, 1.09 to 3.20) for three or more classes. Compared with one dispensing date, the ORs were 2.11 (95% CI, 1.15 to 3.90) for two to three dates, 2.38 (95% CI, 1.32 to 4.29) for four to eight dates, and 2.85 (95% CI, 1.50 to 5.41) for nine or more dates.

In the sensitivity analysis requiring at least five susceptibility categories observed at both cultures, 125 of 558 baseline-negative pairs crossed the recomputed threshold. The global *P*-values were.034 for any ACH-recorded dispensing,.0054 for number of classes, and.0049 for number of days with a dispensing record. The persistence of these associations under a shared-panel definition did not support changing susceptibility-panel composition as their sole explanation. These exploratory findings describe a study-defined seven-category outcome and should not be interpreted as a consensus multidrug-resistance analysis or as treatment-duration effects.

## Discussion

### Principal findings

In this cohort of recurrent urinary GNB, interim class-matched ACH-recorded dispensing was associated with higher adjusted odds of an S→NS transition for ESC, DTR, Pip-Taz, and fluoroquinolones, but not for TMP-SMX, amino-BLI, or carbapenems. Matched dispensing was also associated with lower odds of an NS→S transition, reaching significance only for Pip-Taz. The ESC and fluoroquinolone estimates were directionally consistent across inverse probability weighting, protopathic-buffer variation, and E-value analyses; however, these sensitivity analyses do not eliminate residual confounding, exposure misclassification, or selection bias. Two pragmatic null-covariable estimates reached nominal significance, so residual confounding remains a live alternative explanation for the observed contrasts. These estimates differ slightly from earlier internal values because the comorbidity count was recomputed leave-one-out for each test.

### Interpretation in the context of recent literature

The present 1,274-pair cohort is nested within the broader Aseer Central Hospital microbiology surveillance source used by Shabi et al. (2026), but it adds patient-level same-species pairing and pharmacy linkage and addresses a distinct longitudinal question. The findings extend that population-level surveillance, where carbapenem resistance and DTR among *Klebsiella* species rose steeply over the study period, and are consistent with regional reports of expanding ESC and carbapenem resistance among GNB across Saudi Arabia and the Arabian Peninsula (Alkhelaiwi et al., 2026; Zowawi et al., 2013). They also align with recent longitudinal analyses outside the region: in a national US cohort of more than 390,000 urinary Enterobacterales cultures, a history of class-specific antibiotic exposure was, after prior class-specific resistance, the strongest predictor of subsequent non-susceptibility, with the largest effect for fluoroquinolones and little contribution from exposure to other classes (Brintz et al., 2024). The association between class-matched exposure and subsequent non-susceptibility is biologically coherent with antibiotic selection pressure and aligns with the rationale underpinning international stewardship frameworks (World Health Organization, 2015, 2023). That the strongest signals involved ESC and fluoroquinolones, agents used heavily in empirical therapy, further supports a selection interpretation rather than chance and parallels the high fluoroquinolone non-susceptibility and frequent same-species recurrence reported in community-onset ESC-resistant urinary infection (Zhang et al., 2023). Consistent with our extended-spectrum cephalosporin non-susceptibility signal, Tande et al. reported that greater cumulative antibiotic exposure during the preceding year was associated with ceftriaxone-resistant Enterobacterales among adults with urinary-source Gram-negative bloodstream infection (Tande et al., 2025). Notably, carbapenem exposure was not associated with a subsequent carbapenem S→NS transition, which may reflect the smaller number of carbapenem S→NS events and the predominance of non-exposure pathways to carbapenem resistance, including clonal transmission of carbapenemase-producing organisms documented regionally (Alkhelaiwi et al., 2026; Shabi et al., 2026). A recent longitudinal genomic study of patients with long-term urinary catheters found that strain replacement was uncommon, with 18 of 20 participants retaining the same *Staphylococcus aureus* sequence type over time. This finding supports within-host persistence rather than repeated acquisition, although its generalizability to other urinary species and non-catheter-associated infections remains uncertain (Duran Ramirez et al., 2026). Within-host genomic analysis of recalcitrant bloodstream infection at other body sites points the same way, with persistence attributed to adaptation within a retained strain rather than to replacement by a newly acquired one (Simar et al., 2025). The exploratory cross-class associations for Pip-Taz and ESC exposure are compatible with co-selection but remain hypothesis-generating given multiple testing. Brintz et al. (2024) developed prediction models for urinary Enterobacterales, whereas Tande et al. (2025) studied urinary-source bloodstream infection; these studies provide context but are not direct comparators for the present paired-culture analysis.

### Stewardship and clinical implications

Clinically, these observational results do not estimate the causal effect of prescribing or prove that changing a specific prescription would prevent a later transition. They, nevertheless, support evaluating prior class-matched dispensing when designing surveillance and stewardship studies and reinforce established guidance to avoid unnecessary treatment of asymptomatic bacteriuria (Nicolle et al., 2019) and to reserve last-line agents for confirmed infection (Kadri et al., 2018; Tamma et al., 2022). For DTR, the matched exposure was any systemic antibiotic so that estimate speaks to overall antibiotic pressure rather than to any particular class. Read together, the any-antibiotic and multi-class results bear on cumulative antibiotic pressure rather than on any single agent. Any ACH-recorded dispensing was not clearly associated with crossing the study-defined multi-class non-susceptibility threshold (OR, 1.40; 95% CI, 0.91 to 2.16), whereas among pairs with recorded dispensing both the number of distinct classes and the number of days with a dispensing record were associated with that transition, with odds 1.86 to 2.85 times those of the lowest category. A graded pattern of this kind is compatible with accumulating selection pressure, but exposure categories were not randomized, patients who receive more classes over more days differ systematically from those who do not, and the outcome is a study-defined seven-category construct rather than a consensus multidrug-resistance definition (Magiorakos et al., 2012). These associations are therefore hypothesis-generating and do not establish that narrowing the breadth or shortening the duration of therapy would reduce multi-class non-susceptibility.

### Surveillance and methodological implications

From a surveillance perspective, these data add individual-level, longitudinal evidence to a literature dominated by cross-sectional reporting (World Health Organization Regional Office for the Eastern Mediterranean, n.d; Zowawi, 2016) and illustrate how routinely collected microbiology and pharmacy records can be linked to monitor resistance dynamics and prioritize local stewardship in a high-burden region (Alkhelaiwi et al., 2026; Shabi et al., 2026; Zowawi et al., 2013). This approach responds directly to calls from regional surveillance, which, while documenting high ESC and carbapenem non-susceptibility among urinary Enterobacterales in Saudi Arabia, have largely been cross-sectional and have explicitly identified longitudinal analysis of within-patient susceptibility as a priority for guiding stewardship (Sawan et al., 2025).

### Strengths and limitations

Strengths include the paired within-patient, same-species structure, prespecified feasibility gating, transparent direction-specific transition definitions, and multiple confounding-adjustment and sensitivity analyses, including inverse probability weighting, E-values, penalized-likelihood models, and exploratory pragmatic null-covariable diagnostics.

Several limitations warrant consideration. This was a single-center, retrospective study at one tertiary referral center in southern Saudi Arabia, limiting generalizability, and residual confounding and information bias cannot be excluded despite multivariable adjustment and sensitivity analyses; because the direction of any such bias cannot be established from these data, the reported odds ratios may either overstate or understate the association between class-matched dispensing and a susceptibility transition. The pragmatic null-covariable results were mixed: documented diabetes was nominally associated with the Pip-Taz S→NS transition (OR, 0.49; *P* = .008) and the fluoroquinolone NS→S transition (OR, 1.99; *P* = .015), while 11 other estimates departed from the descriptive odds-ratio range without *P* <.05. These findings do not prove confounding, but they demonstrate that residual confounding or model misspecification remains plausible and preclude causal interpretation. Because the cohort was culture-based rather than a symptom-defined urinary tract infection cohort, positive cultures may represent asymptomatic bacteriuria, colonization, or contamination, and urinary symptoms, pyuria, and colony-count data were unavailable; eligibility required a second same-species culture, restricting inference to recurrent or persistent urinary GNB. Without molecular typing, the analysis cannot distinguish within-strain selection, replacement by another strain of the same species, or changing detection of coexisting subpopulations.

Institutional dispensing, not total receipt or adherence: The exposure estimand was ACH-recorded dispensing. The record did not capture prescriptions filled outside ACH and did not establish administration, ingestion, or adherence. Consequently, absence of a qualifying record did not establish absence of antibiotic receipt. If dispensing obtained outside ACH was missed to the same degree among pairs that later did and did not undergo a susceptibility transition, attenuation toward the null would generally be expected. If instead the probability of a missed dispensing record differed between those two outcome groups, the bias could run in either direction. No external dispensing registry or validation sample was available to distinguish these situations, so the magnitude and direction of this bias cannot be identified from these data. No regional dispensing registry, insurance claims source, or validation subsample was available to quantify the proportion of antibiotic courses obtained outside ACH, so the extent of exposure under-ascertainment could not be estimated.

Clinical indication, dose, administration, defined daily doses, prescribing care setting, catheterization, length of stay, source control, adequacy of empirical coverage, and antibiotic history preceding the first culture were not characterized, preventing dose–response or indication-specific interpretation and leaving substantial potential for confounding by indication and care intensity. Physiologic severity scores (e.g., SOFA, APACHE II) were unavailable; adjustment used ICU status and a documented comorbidity count that is not a validated index such as the Elixhauser point system (van Walraven et al., 2009) and is not established as uniformly pre-first-culture because its source timing was mixed. Because patients who are more acutely ill receive both more antibiotics and more cultures, incomplete adjustment for severity would be expected to bias the S→NS odds ratios away from the null and the NS→S odds ratios below 1.0, so these estimates are more likely to be overstated than understated. Susceptibility used time-specific CLSI breakpoints without raw-MIC reinterpretation, identification used VITEK 2 without MALDI-TOF MS, and resistance mechanisms were not molecularly characterized, and genotype–phenotype discordance is well documented among urinary Enterobacterales (Deka et al., 2026); intermediate and resistant results were combined as non-susceptible. Each of these measurement choices reduces phenotype resolution and could bias the odds ratios in either direction, most plausibly toward the null. Analysis of only the first pharmacy-observable pair per patient-species may underestimate cumulative exposure effects.

## Conclusion

In this regional cohort of recurrent urinary GNB, interim class-matched ACH-recorded dispensing was associated with higher odds of an S→NS transition for extended-spectrum cephalosporins, fluoroquinolones, piperacillin–tazobactam, and difficult-to-treat resistance and with lower odds of an NS→S transition for piperacillin–tazobactam. These culture-based observational associations do not establish causation or strain-level emergence or replacement. They show that paired microbiology and pharmacy records can identify hypotheses about susceptibility transitions for prospective, molecular, and multicenter evaluation.

## Data Availability

The raw data supporting the conclusions of this article will be made available by the authors, without undue reservation.

## References

[B1] Abdalla J. S. Albarrak M. Alhasawi A. Al-Musawi T. Alraddadi B. M. Al Wali W. . (2023). Narrative review of the epidemiology of hospital-acquired pneumonia and ventilator-associated pneumonia in Gulf Cooperation Council countries. Infect. Dis. Ther. 12, 1741–1773. doi: 10.1007/s40121-023-00834-w 37389707 PMC10390449

[B2] Adeolu M. Alnajar S. Naushad S. Gupta R. S. (2016). Genome-based phylogeny and taxonomy of the ‘Enterobacteriales’: proposal for Enterobacterales ord. nov. divided into the families Enterobacteriaceae, Erwiniaceae fam. nov. Pectobacteriaceae fam. nov. Yersiniaceae fam. nov. Hafniaceae fam. nov. Morganellaceae fam. nov. and Budviciaceae fam. nov. Int. J. Syst. Evol. Microbiol. 66, 5575–5599. doi: 10.1099/ijsem.0.001485 27620848

[B3] Alkhelaiwi F. M. Somily A. M. Alahmadi R. M. Awadalla M. Albarrag A. M. Alosaimi B. . (2026). Resistance, virulence, and molecular epidemiology of carbapenem-resistant Klebsiella pneumoniae causing bloodstream infections in Saudi Arabia. Microorganisms 14, 333. doi: 10.3390/microorganisms14020333 41753620 PMC12943425

[B4] Austin P. C. Stuart E. A. (2015). Moving towards best practice when using inverse probability of treatment weighting (IPTW) using the propensity score to estimate causal treatment effects in observational studies. Stat. Med. 34, 3661–3679. doi: 10.1002/sim.6607 26238958 PMC4626409

[B5] Benchimol E. I. Smeeth L. Guttmann A. Harron K. Moher D. Petersen I. . (2015). The REporting of studies Conducted using Observational Routinely-collected health Data (RECORD) statement. PloS Med. 12, e1001885. doi: 10.1371/journal.pmed.1001885 26440803 PMC4595218

[B6] Benjamini Y. Hochberg Y. (1995). Controlling the false discovery rate: a practical and powerful approach to multiple testing. J. R. Stat. Soc Ser. B. Stat. Methodol. 57, 289–300. doi: 10.1111/j.2517-6161.1995.tb02031.x40046247

[B7] Brintz B. J. Madaras-Kelly K. Nevers M. K. Echevarria K. L. Goetz M. B. Samore M. H. (2024). Predicting antibiotic resistance in Enterobacterales to support optimal empiric treatment of urinary tract infections in outpatient veterans. Antimicrob. Steward. Healthc. Epidemiol. 4, e118. doi: 10.1017/ash.2024.377 39257425 PMC11384162

[B8] Clinical and Laboratory Standards Institute (2013–2024a). Performance Standards for Antimicrobial Susceptibility Testing. Annual CLSI Supplement M100 Editions in Force During the Study Period, From M100-S23 (2013) Through M100, 34th Ed. (2024) (Wayne, PA: Clinical and Laboratory Standards Institute).

[B9] Cole S. R. Hernán M. A. (2008). Constructing inverse probability weights for marginal structural models. Am. J. Epidemiol. 168, 656–664. doi: 10.1093/aje/kwn164 18682488 PMC2732954

[B10] Deka N. Brauer A. L. Connerton K. Hanson B. M. Walker J. N. Armbruster C. E. (2026). Pangenome analysis of Proteus mirabilis reveals lineage-specific antimicrobial resistance profiles and discordant genotype-phenotype correlations. Antimicrob. Agents Chemother. 70, e0176825. doi: 10.1128/aac.01768-25 42210697 PMC13321803

[B11] Duran Ramirez J. M. Armbruster C. E. Hanson B. M. Kramer C. Baibhav D. Bhargava S. . (2026). Genomics reveal Staphylococcus aureus persists during long-term urinary catheterization despite antimicrobial therapy and catheter exchanges. Nat. Commun. 17, 1328. doi: 10.1038/s41467-025-68081-w 41530149 PMC12873216

[B12] El-Saed A. Al-Jardani A. Althaqafi A. Alansari H. Alsalman J. Al Maskari Z. . (2016). Ventilator-associated pneumonia rates in critical care units in 3 Arabian Gulf countries: a 6-year surveillance study. Am. J. Infect. Control 44, 794–798. doi: 10.1016/j.ajic.2016.01.042 27040565

[B13] Firth D. (1993). Bias reduction of maximum likelihood estimates. Biometrika 80, 27–38. doi: 10.1093/biomet/80.1.27

[B14] Heinze G. Schemper M. (2002). A solution to the problem of separation in logistic regression. Stat. Med. 21, 2409–2417. doi: 10.1002/sim.1047 12210625

[B15] Kadri S. S. Adjemian J. Lai Y. L. Spaulding A. B. Ricotta E. Prevots D. R. . (2018). Difficult-to-treat resistance in gram-negative bacteremia at 173 US hospitals: retrospective cohort analysis of prevalence, predictors, and outcome of resistance to all first-line agents. Clin. Infect. Dis. 67, 1803–1814. doi: 10.1093/cid/ciy378 30052813 PMC6260171

[B16] Langan S. M. Schmidt S. A. Wing K. Ehrenstein V. Nicholls S. G. Filion K. B. . (2018). The reporting of studies conducted using observational routinely collected health data statement for pharmacoepidemiology (RECORD-PE). BMJ 363, k3532. doi: 10.1136/bmj.k3532 30429167 PMC6234471

[B17] Linden A. Mathur M. B. VanderWeele T. J. (2020). Conducting sensitivity analysis for unmeasured confounding in observational studies using E-values: the evalue package. Stata J. 20, 162–175. doi: 10.1177/1536867X20909696

[B18] Lipsitch M. Tchetgen Tchetgen E. Cohen T. (2010). Negative controls: a tool for detecting confounding and bias in observational studies. Epidemiology 21, 383–388. doi: 10.1097/EDE.0b013e3181d61eeb 20335814 PMC3053408

[B19] Magiorakos A. P. Srinivasan A. Carey R. B. Carmeli Y. Falagas M. E. Giske C. G. . (2012). Multidrug-resistant, extensively drug-resistant and pandrug-resistant bacteria: an international expert proposal for interim standard definitions for acquired resistance. Clin. Microbiol. Infect. 18, 268–281. doi: 10.1111/j.1469-0691.2011.03570.x 21793988

[B20] Nicolle L. E. Gupta K. Bradley S. F. Colgan R. DeMuri G. P. Drekonja D. . (2019). Clinical practice guideline for the management of asymptomatic bacteriuria: 2019 update by the Infectious Diseases Society of America. Clin. Infect. Dis. 68, e83–e110. doi: 10.1093/cid/ciy1121 30895288

[B21] Parte A. C. Sardà Carbasse J. Meier-Kolthoff J. P. Reimer L. C. Göker M. (2020). List of Prokaryotic names with Standing in Nomenclature (LPSN) moves to the DSMZ. Int. J. Syst. Evol. Microbiol. 70, 5607–5612. doi: 10.1099/ijsem.0.004332 32701423 PMC7723251

[B22] Sawan A. A. Alghamdi N. S. Alzahrani S. A. Alharbi M. S. Alabdulkareem N. Alnufaily D. A. . (2025). Urinary tract infections caused by extended-spectrum beta-lactamase-producing and carbapenem-resistant Enterobacterales in Saudi Arabia: impact of catheterization. Med. (Kaunas) 61, 1907. doi: 10.3390/medicina61111907 41303744 PMC12654839

[B23] Shabi Y. Algarni A. M. Binkhamis K. Al Bshabshe A. Alazraqi T. Mohammed S. A. . (2026). Hospital-based surveillance of Klebsiella pneumoniae and other Klebsiella species in southern Saudi Arabia, (2012–2024): escalating carbapenem and DTR resistance with species-specific phenotypes and genotypes. Infect. Drug Resist. 19, 1–13. doi: 10.2147/IDR.S554543 41890238 PMC13015957

[B24] Simar S. R. Tran T. T. Rydell K. B. Atterstrom R. L. Sahasrabhojane P. V. Dinh A. Q. . (2025). Clinical and genomic characterization of recalcitrant enterococcal bacteremia: a multicenter prospective cohort study (VENOUS). J. Infect. Dis. 232, 1351–1364. doi: 10.1093/infdis/jiaf358 40629152 PMC12718003

[B25] Tacconelli E. Cataldo M. A. Paul M. Leibovici L. Kluytmans J. Schröder W. . (2016). STROBE-AMS: recommendations to optimise reporting of epidemiological studies on antimicrobial resistance and informing improvement in antimicrobial stewardship. BMJ Open 6, e010134. doi: 10.1136/bmjopen-2015-010134 26895985 PMC4762075

[B26] Tamma P. D. Aitken S. L. Bonomo R. A. Mathers A. J. van Duin D. Clancy C. J. (2022). Infectious Diseases Society of America 2022 guidance on the treatment of extended-spectrum β-lactamase producing Enterobacterales (ESBL-E), carbapenem-resistant Enterobacterales (CRE), and Pseudomonas aeruginosa with difficult-to-treat resistance (DTR-P. aeruginosa). Clin. Infect. Dis. 75, 187–212. doi: 10.1093/cid/ciac268 35439291 PMC9890506

[B27] Tande A. J. Stevens R. W. Ilges D. Lahr B. D. Challener D. W. (2025). Cumulative antibiotic exposure is associated with ceftriaxone resistance among patients with urinary source gram-negative bloodstream infection. Clin. Infect. Dis. 81, 871–878. doi: 10.1093/cid/ciaf315 40518932

[B28] Van Calster B. McLernon D. J. van Smeden M. Wynants L. Steyerberg E. W. (2019). Calibration: the Achilles heel of predictive analytics. BMC Med. 17, 230. doi: 10.1186/s12916-019-1466-7 31842878 PMC6912996

[B29] VanderWeele T. J. (2017). On a square-root transformation of the odds ratio for a common outcome. Epidemiology 28, e58–e60. doi: 10.1097/EDE.0000000000000733 28816709 PMC5617805

[B30] VanderWeele T. J. Ding P. (2017). Sensitivity analysis in observational research: introducing the E-value. Ann. Intern. Med. 167, 268–274. doi: 10.7326/M16-2607 28693043

[B31] van Smeden M. de Groot J. A. H. Moons K. G. M. Collins G. S. Altman D. G. Eijkemans M. J. C. . (2016). No rationale for 1 variable per 10 events criterion for binary logistic regression analysis. BMC Med. Res. Methodol. 16, 163. doi: 10.1186/s12874-016-0267-3 27881078 PMC5122171

[B32] van Walraven C. Austin P. C. Jennings A. Quan H. Forster A. J. (2009). A modification of the Elixhauser comorbidity measures into a point system for hospital death using administrative data. Med. Care 47, 626–633. doi: 10.1097/MLR.0b013e31819432e5 19433995

[B33] von Elm E. Altman D. G. Egger M. Pocock S. J. Gøtzsche P. C. Vandenbroucke J. P. (2007). The Strengthening the Reporting of Observational Studies in Epidemiology (STROBE) statement: guidelines for reporting observational studies. Lancet 370, 1453–1457. doi: 10.1016/S0140-6736(07)61602-X 18064739

[B34] World Health Organization (2015). Global Action Plan on Antimicrobial Resistance (Geneva: World Health Organization).

[B35] World Health Organization (2023). Antimicrobial Resistance. Available online at: https://www.who.int/news-room/fact-sheets/detail/antimicrobial-resistance (Accessed May 18, 2026).

[B36] World Health Organization Regional Office for the Eastern Mediterranean . Antimicrobial Resistance in the Region. Available online at: https://www.emro.who.int/health-topics/drug-resistance/regional-situation.html (Accessed May 18, 2026).

[B37] Zhang H. L. Perez R. Krishnan J. Lautenbach E. Anderson D. J. (2023). Risk factors for recurrence of community-onset urinary tract infections caused by extended-spectrum cephalosporin-resistant Enterobacterales. Open Forum Infect. Dis. 10, ofad561. doi: 10.1093/ofid/ofad561 38130594 PMC10733196

[B38] Zowawi H. M. (2016). Antimicrobial resistance in Saudi Arabia: an urgent call for an immediate action. Saudi Med. J. 37, 935–940. doi: 10.15537/smj.2016.9.16139 27570847 PMC5039611

[B39] Zowawi H. M. Balkhy H. H. Walsh T. R. Paterson D. L. (2013). β-Lactamase production in key gram-negative pathogen isolates from the Arabian Peninsula. Clin. Microbiol. Rev. 26, 361–380. doi: 10.1128/CMR.00096-12 23824364 PMC3719487

