# Supplementary material for: Class-matched antibiotic dispensing and susceptibility transitions in recurrent urinary gram-negative bacilli
Source: Front Cell Infect Microbiol. 2026 Sep 28;16:1897524. doi: 10.3389/fcimb.2026.1897524 (PMC13654793; doi:10.3389/fcimb.2026.1897524)
Supplement: Supplementary file 1 [file Table1.docx]

**Supplementary Material**

*Class-Matched Antibiotic Dispensing and Susceptibility Transitions in Recurrent Urinary Gram-Negative Bacilli*

This file replaces the previous Supplementary Materials and eMETHODS documents. Terminology, breakpoint citation and the comorbidity covariable have been brought into line with the revised manuscript: susceptibility transitions are described throughout as S→NS and NS→S, breakpoints are cited as the CLSI editions in force during 2013–2024, and the Elixhauser index has been replaced by the documented comorbidity count.

**eMethods**

**1. Study Population**

**Pair derivation.**

Within each patient-species stratum, cultures were ordered by culture reception date with sample number as the tiebreaker, and same-patient, same-species cultures collected less than 14 days apart were collapsed to the earliest record. Candidate pairs were formed by forward-looking selection of the next same-patient, same-species culture, and a pair was eligible when the inter-culture interval (second-culture date minus first-culture date) fell between 14 and 365 days. A pair was classified as pharmacy-observable if the patient had at least one Aseer Central Hospital (ACH) dispensing record on or before the first culture; pairs without a qualifying record were retained for flow accounting only. Descriptive analyses retained the first pharmacy-observable pair per patient; phenotype-specific regression analyses retained the first pharmacy-observable pair per patient-species combination. Same-species pairing did not establish strain identity. Without molecular typing, a transition may reflect selection within a persisting strain, replacement by another strain of the same species, or changing detection of coexisting subpopulations.

**Between-culture exposure.**

Among pharmacy-observable pairs, interim exposure was defined from the ACH dispensing record using a deterministic date window anchored on the pair dates. A dispensing event qualified when it occurred after the first culture and before the 3-day interval immediately preceding the second culture (3-day protopathic buffer). Eligible systemic routes were oral, intravenous, intramuscular and combined intravenous/intramuscular administration. Exposure flags were collapsed into phenotype-matched class indicators: fluoroquinolones, extended-spectrum cephalosporins (ESC), carbapenems, piperacillin-tazobactam (Pip-Taz), trimethoprim-sulfamethoxazole (TMP-SMX) and aminopenicillins with beta-lactamase inhibitors (Amino-BLI). Because difficult-to-treat resistance (DTR) is a composite phenotype with no single cognate class, its matched exposure was any ACH-recorded systemic antibiotic, making that contrast any versus none. The exposure estimand is institutional dispensing, not total antibiotic receipt: the record does not capture prescriptions filled outside ACH and does not establish administration, ingestion or adherence.

**2. Laboratory Methods**

Species identification and antimicrobial susceptibility testing were performed under routine institutional microbiology workflows using the VITEK 2 automated system (bioMérieux, Marcy-l’Étoile, France); MALDI-TOF MS was not available during the study period. Minimum inhibitory concentrations were interpreted according to the Clinical and Laboratory Standards Institute (CLSI) breakpoints in effect at the time of testing, that is, the annual M100 supplements in force during the study period from M100-S23 (2013) through M100, 34th edition (2024); raw MIC values were not available for retrospective reinterpretation. Results were harmonised to susceptible versus non-susceptible, with intermediate results grouped with resistant results to reduce misclassification from temporal breakpoint revisions. Organism-drug pairs with intrinsic resistance, absent CLSI interpretive criteria or prespecified non-reportable combinations were suppressed before susceptibility summaries or phenotype derivation and coded as missing rather than susceptible ; the executed rules are held with the analysis code.

Non-susceptibility phenotypes were defined for fluoroquinolones (ciprofloxacin or levofloxacin), ESC restricted to Enterobacterales (cefotaxime, ceftazidime, ceftriaxone or cefepime), carbapenems (ertapenem, imipenem or meropenem; imipenem excluded for Proteeae), Pip-Taz, TMP-SMX, and Amino-BLI (amoxicillin-clavulanate or ampicillin-sulbactam). DTR was defined separately for Enterobacterales, Acinetobacter baumannii and Pseudomonas aeruginosa using criteria adapted from Kadri et al. (2018). Stenotrophomonas maltophilia and other non-fermenting gram-negative bacilli not represented in that framework were excluded from DTR classification.

**3. Statistical Analysis**

**Phenotype feasibility gating.**

Before modelling, a phenotype was retained if the pair dataset contained at least 50 S→NS events overall, at least 10 pairs with matched antibiotic dispensing, and at least 10 S→NS events among matched-exposed pairs. Seven of ten candidate phenotypes met these criteria (Table 2 of the main text).

**Primary S→NS model.**

For each retained phenotype the S→NS odds ratio was estimated from a first-order Markov transition logistic model of second-culture non-susceptibility. The model included baseline susceptibility status, class-matched ACH-recorded dispensing, the inter-culture interval modelled with restricted cubic splines using four knots, and interactions of baseline susceptibility status with exposure and with the interval. The exposure effect among baseline-susceptible pairs yielded the S→NS odds ratio. Adjusted models added age, sex, organism group, intensive care unit status at culture collection, prior culture burden and the documented comorbidity count. Standard errors were clustered at the patient level.

**Direct NS→S models.**

NS→S odds ratios were estimated directly in separate logistic models restricted to baseline-non-susceptible pairs, with susceptibility at the second culture coded as the event. They were not derived by inverting a persistence odds ratio. Within that restricted sample, baseline status and its interactions are constant or collinear and are therefore omitted. Values below 1.0 indicate that class-matched dispensing is associated with lower odds of a return to susceptibility.

**Documented comorbidity count.**

The count is the unweighted sum of 18 documented binary conditions (acute kidney injury, asthma, cancer, chronic kidney disease, chronic liver disease, chronic obstructive pulmonary disease, congestive heart failure, dementia, diabetes, epilepsy, HIV, hypertension, ischaemic heart disease, obesity, peripheral vascular disease, sickle cell disease, systemic lupus erythematosus and stroke). It is not a validated comorbidity index such as the Elixhauser point system. Components were ascertained from admission diagnosis coding, the operative record, the pharmacy record and pathology reports. Admission-derived flags are episode-level, operation and pharmacy evidence was restricted through each culture, and pathology evidence was undated and assigned at the patient level; the count is therefore not uniformly measured before the first culture.

**Penalised likelihood and model performance.**

Firth penalised-likelihood sensitivity models were fitted for both transition directions and all seven phenotypes, using profile penalised-likelihood confidence intervals without cluster-robust variance, and compared with the conventional patient-clustered models, which remained the reference. Model N, transition events, full-model degrees of freedom, events per full-model degree of freedom, c-statistics, Brier scores and calibration intercepts and slopes are summarised in the main text. Optimism was estimated from 500 patient-cluster bootstrap resamples per model.

**Multiplicity.**

Benjamini-Hochberg correction was applied once across the 14 conventional primary transition tests, and separately across the exploratory cross-class tests and across the three primary global tests of the multi-class outcome. A nominal two-sided P < .05 was used before correction.

**Inverse probability weighting.**

As a secondary analysis, stabilised inverse probability weighting was applied for each phenotype. The propensity model regressed the probability of receiving the class-matched exposure on the primary-analysis covariables, baseline susceptibility status at the first culture, the inter-culture interval as a restricted cubic spline, and the interval-by-baseline-susceptibility interaction. Stabilised weights used the marginal exposure probability within the phenotype-specific propensity estimation sample as numerator and the fitted probability from the full propensity model as denominator. Weight means, dispersion and maxima were summarised before and after winsorisation at the 1st and 99th percentiles. Covariable balance before and after weighting was assessed with the absolute standardised mean difference and a prespecified |SMD| < 0.10 criterion.

**Cross-class models, buffer sensitivity, E-values and pragmatic null covariables.**

Forty-nine first-order Markov transition models were fitted using each antibiotic class as exposure and each phenotype as outcome; class-matched contrasts correspond to the primary analysis and cross-class contrasts were treated as exploratory (Figure 5 of the main text). The protopathic buffer was varied from the primary 3 days to 0, 7 and 14 days, and both transition models were refitted at each buffer (eFigure 3). E-values were computed for the point estimate and the confidence bound closer to the null for all 14 conventional primary estimates (eFigure 4); the conversion was keyed to the overall target-transition rate in each phenotype-direction estimation sample, using the odds ratio as an approximation to the risk ratio where that rate was below 15% and the square root of the odds ratio where it was 15% or greater. E-values bound unmeasured confounding only and do not address selection, exposure or outcome misclassification, or model misspecification. Documented hypertension and documented diabetes were entered individually alongside class-matched exposure as pragmatic null covariables, with the documented comorbidity count recomputed leave-one-out for each test so that the model did not condition on a count containing the variable under test. A result was descriptively classified as consistent with the null when the odds ratio lay between 0.80 and 1.25 and P exceeded .05; this descriptive threshold was not an equivalence test.

**Further sensitivity analyses.**

All 14 conventional models were refitted with the documented comorbidity count omitted. Because pathology-derived comorbidity evidence lacked reliable dates, every count component that could have been supported by biopsy evidence was removed and the conventional and weighted models refitted. Finally, the already selected analytic pairs were restricted to those whose first culture linked to a hospital admission episode, without substituting any replacement pair; this restriction changes the target population toward admission-linked care and does not establish present-on-admission status.

**Exploratory multi-class outcome.**

A study-defined seven-category multi-class non-susceptibility outcome was defined as non-susceptibility in at least three of seven observed categories when at least three were tested. Among pairs below that threshold at the first culture, separate patient-clustered logistic models examined any ACH-recorded dispensing, the number of distinct antibiotic classes, and the number of days with a dispensing record. A panel-comparability sensitivity analysis required at least five susceptibility categories to be observed at both cultures and recomputed the outcome from the shared panel only. This outcome is not a consensus multidrug-resistance definition.

**Software.**

Analyses used Stata 19.5 (StataCorp LLC), R 4.6.1 (R Foundation for Statistical Computing) and Python 3.14.6 (Python Software Foundation). Miettinen-Nurminen risk differences used the Stata module rdcii 1.2 with the ridder root finder 1.1.2; E-values used evalue 1.3.0; distinct patient counts used distinct 1.2.1. Firth models used the R package logistf 1.26.1 and c-statistics used pROC 1.19.0.1. Antibiotic-dispensing linkage used pandas 3.0.3 with numpy 2.5.0 and pyarrow 24.0.0.

**eTables**

**eTable 1. Full Comorbidity Profile by Between-culture ACH-recorded Antibiotic Dispensing (N = 1,078 patients)**

| **Comorbidity, n (%)** | **Overall (N = 1,078)** | **Between-culture Antibiotic Exposure** | |
| --- | --- | --- | --- |
|  |  | **No antibiotic (n = 271)** | **Any antibiotic (n = 807)** |
| Acute kidney injury | 21 (1.9) | 3 (1.1) | 18 (2.2) |
| Asthma | 1 (0.1) | 0 (0.0) | 1 (0.1) |
| Atrial fibrillation | 3 (0.3) | 0 (0.0) | 3 (0.4) |
| Cancer (any) | 77 (7.1) | 14 (5.2) | 63 (7.8) |
| Chronic kidney disease | 62 (5.8) | 12 (4.4) | 50 (6.2) |
| Chronic liver disease | 0 (0.0) | 0 (0.0) | 0 (0.0) |
| Chronic obstructive pulmonary disease | 6 (0.6) | 1 (0.4) | 5 (0.6) |
| Congestive heart failure | 16 (1.5) | 1 (0.4) | 15 (1.9) |
| Dementia | 1 (0.1) | 0 (0.0) | 1 (0.1) |
| Diabetes mellitus | 213 (19.8) | 50 (18.5) | 163 (20.2) |
| Epilepsy | 8 (0.7) | 1 (0.4) | 7 (0.9) |
| End-stage kidney disease | 46 (4.3) | 10 (3.7) | 36 (4.5) |
| Hypertension | 380 (35.3) | 90 (33.2) | 290 (35.9) |
| Ischemic heart disease | 4 (0.4) | 1 (0.4) | 3 (0.4) |
| Myocardial infarction | 4 (0.4) | 2 (0.7) | 2 (0.2) |
| Obesity | 5 (0.5) | 1 (0.4) | 4 (0.5) |
| Peripheral vascular disease | 8 (0.7) | 3 (1.1) | 5 (0.6) |
| Rheumatoid arthritis | 1 (0.1) | 0 (0.0) | 1 (0.1) |
| Sickle cell disease | 0 (0.0) | 0 (0.0) | 0 (0.0) |
| Systemic lupus erythematosus | 5 (0.5) | 1 (0.4) | 4 (0.5) |
| Stroke | 42 (3.9) | 6 (2.2) | 36 (4.5) |
| Abbreviations: IQR, interquartile range.  a—First pharmacy-observable culture pair per patient. | | | |

First pharmacy-observable culture pair per patient. Conditions are documented binary flags ascertained from admission diagnosis coding, the operative record, the pharmacy record and pathology reports. Absence of a qualifying record was treated as absence of the condition. The documented comorbidity count used in the models is the unweighted sum of 18 of these conditions and is not a validated comorbidity index; weighted and unweighted Elixhauser indices are no longer reported.

**eTable 2. Species Frequency by Year (N = 1,274 culture pairs)**

| **Species** | **2013** | **2014** | **2015** | **2016** | **2017** | **2018** | **2019** | **2020** | **2021** | **2022** | **2023** | **2024** | **Total** | **%** |
| --- | --- | --- | --- | --- | --- | --- | --- | --- | --- | --- | --- | --- | --- | --- |
| **Enterobacterales** | | | | | | | | | | | | | | |
| *Citrobacter freundii* | — | 1 | 1 | 1 | — | — | — | — | — | — | — | 3 | 6 | 0.5 |
| *Citrobacter sp.* | — | — | — | — | — | 1 | 1 | — | 1 | — | — | 1 | 4 | 0.3 |
| *Enterobacter cloacae* | — | 1 | 2 | 1 | 1 | 1 | 3 | 3 | 2 | 4 | 3 | — | 21 | 1.6 |
| *Enterobacter sp.* | — | — | 1 | — | — | — | — | — | — | — | — | — | 1 | 0.1 |
| *Escherichia coli* | 16 | 40 | 33 | 45 | 42 | 61 | 63 | 46 | 82 | 70 | 94 | 41 | 633 | 49.7 |
| *Klebsiella aerogenes* | — | 1 | 2 | 1 | — | — | — | — | 1 | — | — | 1 | 6 | 0.5 |
| *Klebsiella oxytoca* | — | 1 | 2 | 1 | 1 | — | — | 1 | — | — | 2 | — | 8 | 0.6 |
| *Klebsiella pneumoniae* | 5 | 9 | 7 | 14 | 15 | 23 | 46 | 41 | 44 | 59 | 61 | 21 | 345 | 27.1 |
| *Klebsiella pneumoniae* subsp. *ozaenae* | — | — | — | — | — | 1 | — | — | — | — | — | 1 | 2 | 0.2 |
| *Morganella morganii* | — | 3 | 5 | 1 | 1 | — | 1 | 1 | — | — | 1 | — | 13 | 1.0 |
| *Pluralibacter gergoviae* | — | — | — | — | 1 | — | — | — | — | — | — | — | 1 | 0.1 |
| *Proteus mirabilis* | 1 | 10 | 7 | 6 | 6 | 3 | 13 | 1 | 6 | 8 | 3 | 2 | 66 | 5.2 |
| *Providencia stuartii* | 3 | 3 | 2 | 2 | — | 1 | 3 | 1 | 1 | — | — | — | 16 | 1.3 |
| *Serratia marcescens* | — | 1 | — | — | — | 1 | 1 | 1 | 2 | 1 | 1 | 2 | 10 | 0.8 |
| **Subtotal** | **25** | **70** | **62** | **72** | **67** | **92** | **131** | **95** | **139** | **142** | **165** | **72** | **1,132** | **88.9** |
| **Non-fermenting gram-negative bacilli** | | | | | | | | | | | | | | |
| *Acinetobacter baumannii* | 3 | 3 | 2 | 3 | 3 | 1 | 2 | 1 | 2 | 4 | 6 | 1 | 31 | 2.4 |
| *Pseudomonas aeruginosa* | 4 | 6 | 16 | 13 | 9 | 7 | 14 | 6 | 10 | 11 | 13 | 1 | 110 | 8.6 |
| *Pseudomonas sp.* | — | — | — | — | — | — | — | — | — | 1 | — | — | 1 | 0.1 |
| **Subtotal** | **7** | **9** | **18** | **16** | **12** | **8** | **16** | **7** | **12** | **16** | **19** | **2** | **142** | **11.1** |
| **Total** | **32** | **79** | **80** | **88** | **79** | **100** | **147** | **102** | **151** | **158** | **184** | **74** | **1,274** | **100.0** |
| a—First pharmacy-observable culture pair per patient-species (1,078 patients). Em-dash (—) indicates zero isolates in that year. 2024 is a partial year through 2024-08-27. | | | | | | | | | | | | | | |

First pharmacy-observable culture pair per patient-species.

**eFigure Legends**

**eFigure 1. Transition Dumbbell Plot.**

For each retained phenotype, points show the observed exposed and unexposed transition rates for the S→NS and NS→S directions. Labels report exposed-minus-unexposed risk differences with 95% confidence intervals.

**eFigure 2. Weighted-analysis Diagnostics.**

A, Primary and inverse probability weighted S→NS odds ratios for each retained phenotype. B, Absolute standardised mean differences for each covariable before and after weighting, with the |standardised mean difference| = 0.10 threshold indicated.

**eFigure 3. Buffer Sensitivity Trajectories.**

A, S→NS odds ratios for each retained phenotype after varying the protopathic buffer from 0 to 3, 7 and 14 days. B, Corresponding NS→S odds ratios from the direct models. Filled markers indicate estimates whose 95% confidence intervals exclude 1.0; open markers indicate intervals that include 1.0.

**eFigure 4. E-value Plot.**

A, Point-estimate and confidence-bound E-values for the S→NS estimates of each retained phenotype. B, Corresponding E-values for the NS→S estimates. E-values bound unmeasured confounding only. Values for the five estimates that survived correction across the primary family are those reported in the main text; the remaining nine were computed from the adjusted odds ratios in Table 4 using the same transition-rate-keyed conversion described in the eMethods. An open marker denotes a confidence interval that includes the null, for which the bound E-value is 1.00.

**eReferences**

Austin, P. C., and Stuart, E. A. (2015). Moving towards best practice when using inverse probability of treatment weighting (IPTW) using the propensity score to estimate causal treatment effects in observational studies. Stat. Med. 34, 3661–3679. doi: 10.1002/sim.6607

Benjamini, Y., and Hochberg, Y. (1995). Controlling the false discovery rate: a practical and powerful approach to multiple testing. J. R. Stat. Soc. Series B Stat. Methodol. 57, 289–300. doi: 10.1111/j.2517-6161.1995.tb02031.x

Clinical and Laboratory Standards Institute (2013–2024). Performance Standards for Antimicrobial Susceptibility Testing. Annual CLSI supplement M100 editions in force during the study period, from M100-S23 (2013) through M100, 34th ed. (2024). Wayne, PA: Clinical and Laboratory Standards Institute.

Cole, S. R., and Hernán, M. A. (2008). Constructing inverse probability weights for marginal structural models. Am. J. Epidemiol. 168, 656–664. doi: 10.1093/aje/kwn164

Firth, D. (1993). Bias reduction of maximum likelihood estimates. Biometrika 80, 27–38. doi: 10.1093/biomet/80.1.27

Heinze, G., and Schemper, M. (2002). A solution to the problem of separation in logistic regression. Stat. Med. 21, 2409–2419. doi: 10.1002/sim.1047

Kadri, S. S., Adjemian, J., Lai, Y. L., Spaulding, A. B., Ricotta, E., Prevots, D. R., et al. (2018). Difficult-to-treat resistance in gram-negative bacteremia at 173 US hospitals. Clin. Infect. Dis. 67, 1803–1814. doi: 10.1093/cid/ciy378

Lipsitch, M., Tchetgen Tchetgen, E., and Cohen, T. (2010). Negative controls: a tool for detecting confounding and bias in observational studies. Epidemiology 21, 383–388. doi: 10.1097/EDE.0b013e3181d61eeb

van Walraven, C., Austin, P. C., Jennings, A., Quan, H., and Forster, A. J. (2009). A modification of the Elixhauser comorbidity measures into a point system for hospital death using administrative data. Med. Care 47, 626–633. doi: 10.1097/MLR.0b013e31819432e5

VanderWeele, T. J., and Ding, P. (2017). Sensitivity analysis in observational research: introducing the E-value. Ann. Intern. Med. 167, 268–274. doi: 10.7326/M16-2607
